# Fusion protein condensate formation via coiled‐coil domains

**DOI:** 10.1002/pro.70674

**Published:** 2026-06-12

**Authors:** Om Prakash Narayan, Lu Liu, Kyle Scheller, Carter Humphrey, Jiawei Dong, Juan Guan

**Affiliations:** ^1^ Division of Chemical Biology and Medicinal Chemistry, College of Pharmacy University of Texas at Austin Austin Texas USA; ^2^ Department of Molecular Genetics and Microbiology University of Florida College of Medicine Gainesville Florida USA; ^3^ University of Florida Health Cancer Institute Gainesville Florida USA; ^4^ Biotechnology Department Austin Community College Austin Texas USA

**Keywords:** biomolecular condensates, coiled‐coil domain, fusion‐protein, protein condensates

## Abstract

While recent research shows that biomolecular condensates play important roles in normal cellular processes and diseases, the driving forces in condensate formation are not well understood, especially regarding the role of structured self‐associative protein domains. In this work, we study the contribution of a model structured domain, coiled‐coil domain, in promoting condensate formation of fusion proteins (FPs). Starting from a large set of ~50,000 FPs, we systematically narrowed down to investigate 14 FPs and their corresponding 18 coiled‐coil domains. We showed that all 14 FPs are capable of assembling condensates with high potency. When isolated from the rest of the protein contexts, 11 of the 18 coiled‐coil domains can induce condensation on their own, despite their short length compared to their full‐length counterparts. To understand the differences between coiled‐coils that can drive condensate formation and those that cannot, we developed a “triad‐extension” model and found the condensate‐sufficient coiled‐coil domains have a higher propensity to extend beyond perfectly end‐to‐end matched dimer/oligomer to promote condensate formation.

## 1 INTRODUCTION

Proteins and nucleic acids can self‐assemble into micron‐scale compartments in living cells known as biomolecular condensates (BMCs) (Andre et al., 2025; Banani et al., 2017; Boeynaems et al., 2023; Niu et al., 2023; Zhang et al., 2024). The formation of BMCs creates microenvironments with high concentrations of specific proteins and nucleic acids, leading to more efficient and specific biochemical reactions (Brasnett et al., 2024; Laflamme & Mekhail, 2020; Stoffel et al., 2025). BMCs play an important role in normal physiological processes such as ribosome biogenesis and RNA metabolism (Correll et al., 2019; Pessina et al., 2025). Aberrant BMC assemblies have been implicated in various neurodegenerative diseases and cancers (Nam & Gwon, 2023; Qin et al., 2025; Wood, 2023).

While earlier works identified diverse proteins having the ability to assemble into BMCs (Emenecker et al., 2020; Frazer et al., 2020; Guilhas et al., 2020; Ladouceur et al., 2020; Lawrimore et al., 2021; Lyon et al., 2021; Monterroso et al., 2019; Riback et al., 2017; Wunder & Mueller‐Cajar, 2020; Xie et al., 2021; Xing et al., 2020; Yeong et al., 2020), fusion proteins (FPs) have emerged as an interesting class of proteins to study in the BMC field (Tripathi et al., 2023). FPs, or chimera proteins, occur when a chromosomal rearrangement fuses two partial genes and creates a new protein (Tang et al., 2025). These genetic mutations are found in normal tissues, inheritable diseases, and cancers (Oliver et al., 2019, 2020). Prominent examples of FPs forming BMCs with clear causal links to diseases include EWS‐FLI1 (Boulay et al., 2017; Selig et al., 2025) and EML4‐ALK (Sampson et al., 2021; Tulpule et al., 2021). Further studies revealed shared functions and biological consequences of these fusion protein condensates (FPCs)—the FPCs enrich specific proteins to promote biochemical reactions in certain cellular pathways. Interestingly, EWS‐FLI1 BMC assembly is driven by an intrinsically disordered region (IDR) (Boulay et al., 2017) whereas a structured coiled‐coil domain (CCD) is critical for EML4‐ALK BMC formation (Scheller et al., 2025).

While significant progress has been made in systematically defining the condensate landscape of fusion oncoproteins (Tripathi et al., 2023), the domains responsible for assembling FPCs, particularly structured self‐associative domains such as coiled‐coil domains, remain largely uncharacterized. Moreover, whether coiled‐coil domains alone are sufficient to drive condensate formation, and what sequence features distinguish condensate‐promoting from condensate‐deficient coiled‐coils, has not been addressed (Mittag & Ansari, 2021; Tripathi et al., 2023). In particular, although IDRs, prion‐like domains (PLDs), and low‐complexity domains (LCDs) are demonstrated to promote BMC assemblies and have been shown to be enriched in FPs (Tripathi et al., 2023), the contribution of structured self‐associative domains to FPC assembly is less studied.

Recent studies have highlighted the pivotal role of protein–protein interactions between structured domains as an alternative mechanism driving biomolecular condensate formation (Hess & Joseph, 2025). In contrast to IDRs, structured domains often possess well‐defined and biophysically characterized protein interaction interfaces, which are amenable to rational design and optimization through protein engineering strategies. Among these structured domains, the coiled‐coil domain has emerged as a particularly influential motif due to its self‐associative properties and the precise mapping of interacting residues at its interface (He et al., 2025; Ramirez et al., 2024; Truebestein & Leonard, 2016). This domain has been implicated in mediating protein phase separation events in both physiological and pathological contexts including coiled‐coil‐dependent phase separation of TAR DNA‐binding protein 43 (TDP‐43), a non‐FP whose alpha‐helical region modulates its liquid–liquid phase separation, and the oncogenic FP EML4‐ALK, where the coiled‐coil domain is critical for condensate formation (Conicella et al., 2020; Scheller et al., 2025). The characteristic heptad repeat architecture of coiled‐coils enables tunable interaction strength via modulation of coil length, while specific residues at defined positions within the heptad can be strategically altered to fine‐tune binding affinity and specificity (Burkhard et al., 2002; Lebar et al., 2020). Moreover, naturally occurring sequence variants offer a rich repertoire for engineering orthogonal and modular interaction pairs. These unique structural and functional attributes have positioned coiled‐coil domains as highly attractive scaffolds for synthetic biology and protein engineering applications in the study and manipulation of BMCs (Qian et al., 2022; Reinkemeier & Lemke, 2021).

In this work, we use coiled‐coil domains as a model structured self‐associative domain to systematically investigate its contribution in condensate formation of naturally occurring FPs. We reasoned that coiled‐coil domains can initiate oligomeric seeds of FPs which promote BMC formation. We employ a streamlined molecular cloning strategy that scarlessly stitches two gene fragments to efficiently generate FPs. Additionally, quantitative fluorescence imaging is used to pinpoint the CCD contribution in FPCs. We further developed a model that incorporated stable triad formation to account for protein condensation beyond the classical coiled‐coil homo‐oligomers.

## 2 RESULTS AND DISCUSSION

### 2.1 Identification of candidate fusion proteins containing coiled‐coil domains

**FIGURE 1:**
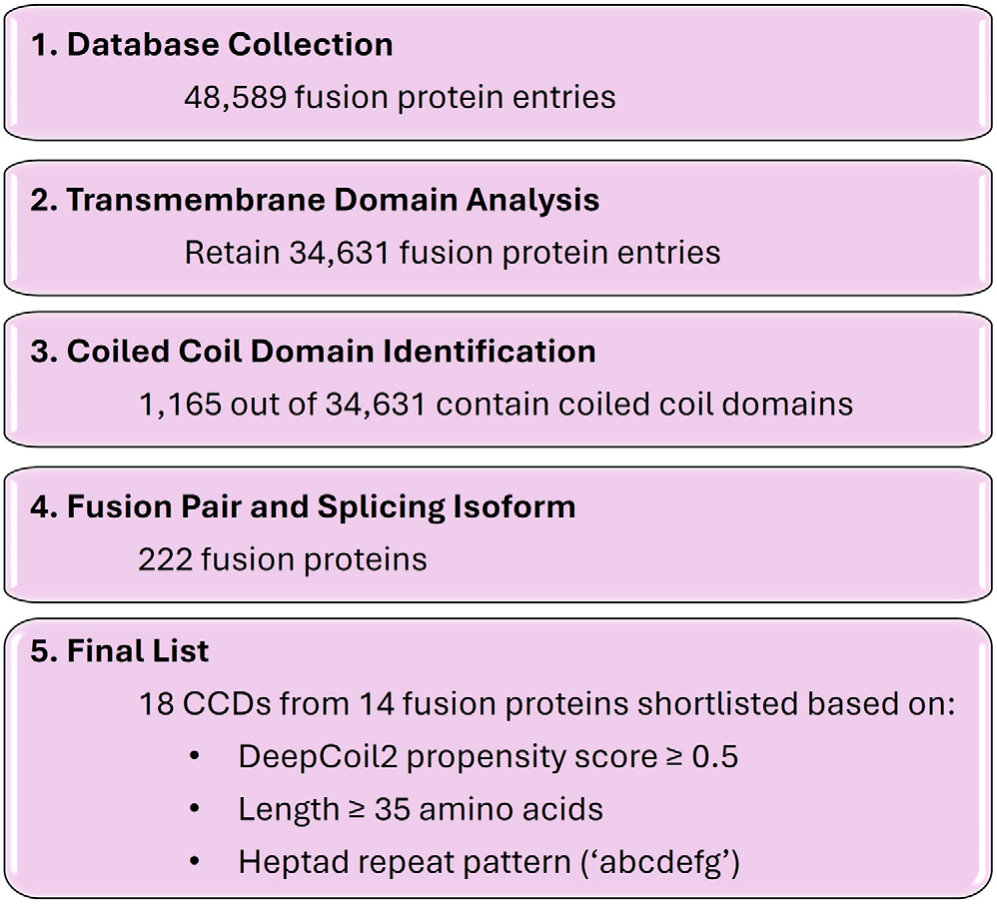
Workflow for the identification of candidate fusion proteins (FPs) containing coiled‐coil domains (CCDs). A large database of fusion proteins was processed by transmembrane domain analysis, CCD prediction to a final set of 14 FPs and correspondingly 18 CCDs for further testing.

The workflow to identify candidate FPs is outlined in Figure 1. A comprehensive list of 48,589 naturally occurring FPs was compiled from a publicly available database (Souza et al., 2025; Tomczak et al., 2015). Because BMCs are membrane‐less subcellular compartments, we retained 34,631 proteins that do not have transmembrane domains via TransMembrane Hidden Markov Model (TMHMM) algorithm (Krogh et al., 2001), a hidden Markov model‐based method for transmembrane helix prediction. Since the focus of this study is to evaluate the role of CCDs in FP condensate formation, we further identified 1165 FPs containing CCDs based on the prediction by DeepCoil2 (https://github.com/labstructbioinf/DeepCoil), a deep‐learning‐based predictor of coiled‐coil regions (Ludwiczak et al., 2019). From this pool, 222 unique fusion pairs were identified when considering splicing isoforms. We prioritized 14 FPs and their corresponding 18 CCDs for downstream experimental characterization by first identifying CCDs within the FPs based on sequence‐based coiled‐coil prediction criteria, including CCD length and DeepCoil2 probability score, and then further evaluated the predicted CCDs using AlphaFold2 and Socket2 to assess coiled‐coil architecture and register assignment. The 14 FPs are CCDC6‐PCBD1, CXCL16‐TEKT1, DVL2‐TFE3, GYS1‐HOMER3, LAMA4‐PSAP, MED1‐KRT222, NSFL1C‐HOOK2, PAX8‐NFE2L2, PCBP4‐POC1A, PDZRN3‐FOXP1, SPAG9‐SKAP1, STRN3‐METTL3, TRIM27‐PTPRR, and VPS37B‐MPHOSPH9 (Table S1). Note that the selection process produced a tractable list of candidate CCDs for further analysis without implying that these predicted CCDs are structurally more robust. In short, the computational analysis described here represents a targeted approach designed to generate a curated list of FPs likely to harbor coiled‐coil‐mediated interactions that contribute to condensate formation.

### 2.2 Full‐length fusion proteins containing coiled‐coil domains form condensates

**FIGURE 2:**
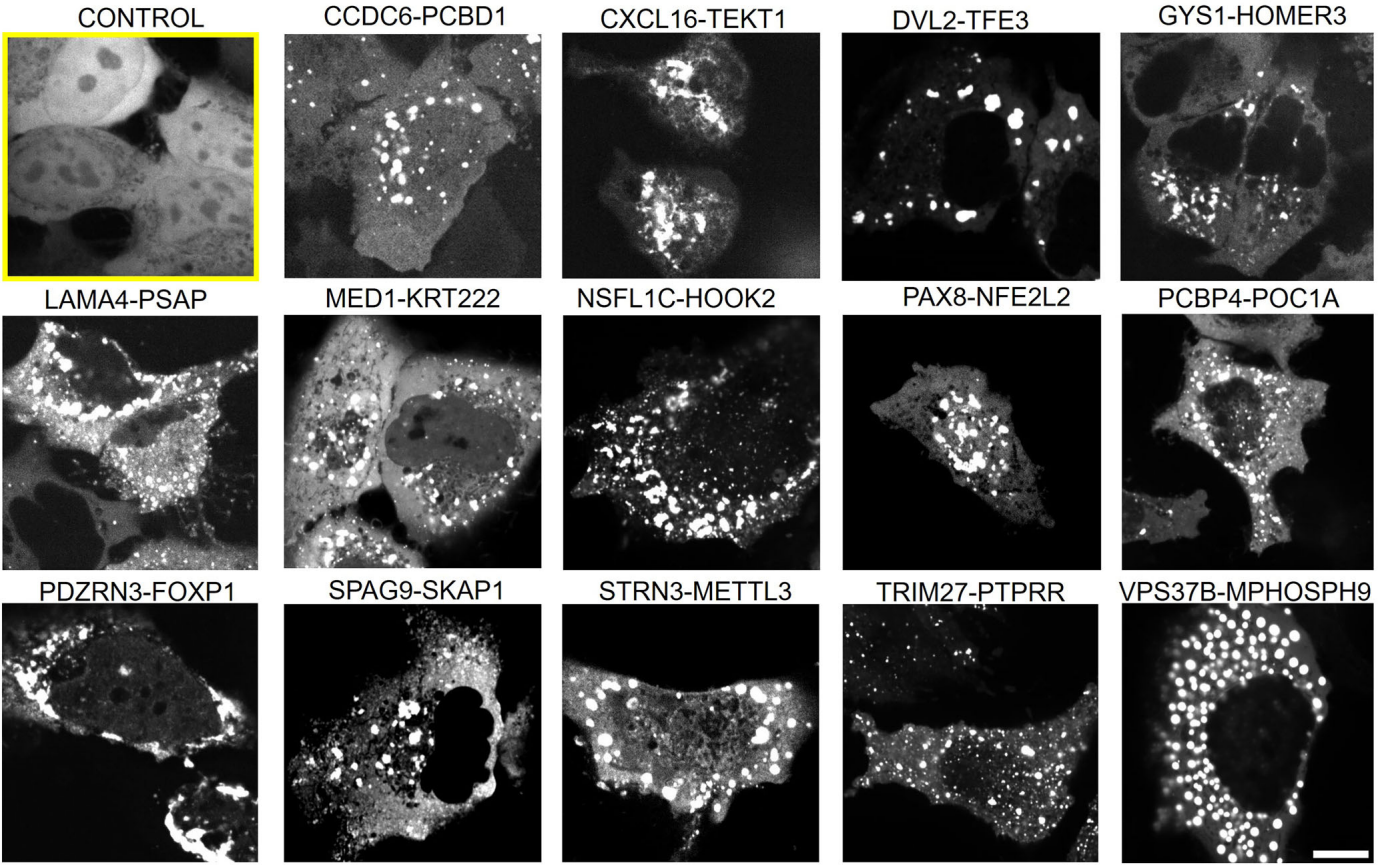
Coiled‐coil domains (CCD)‐containing fusion proteins form condensates in U2OS cells. Bright puncta indicate condensate formation. Control group (highlighted yellow box) expressing mEGFP alone showed diffuse distribution without condensates. Scale bar: 10 μm.

To test the ability of these FPs to form condensates, we directly imaged them with an monomeric enhanced green fluorescence protein (mEGFP) tag in cells with fluorescence microscopy. Strikingly, we observed that all 14 CCD‐containing FPs formed numerous condensates within cells (Figure 2). This observation of prevalent FP condensation is reproducibly seen in multiple cell lines (Figure S1). A closer inspection of the images shows that most FPCs assemble in the cytoplasm. Even though some of the parental proteins are typically located in the nucleus, the FPs lose nuclear localization and are instead found in the cytoplasm. This shift may result from disruption or loss of nuclear localization signals (NLS) upon fusion (Table S5).

Note that although CCD domains were implicated in prior studies to promote FP dimerization or oligomerization, their connection to biomolecular condensate formation remains unclear. Furthermore, while more examples of FPs forming condensates have been identified, those were not discovered systematically with a shared structured folded protein motif. Our imaging results indicate that many FPs with CCD domains are capable of forming BMCs.

**FIGURE 3:**
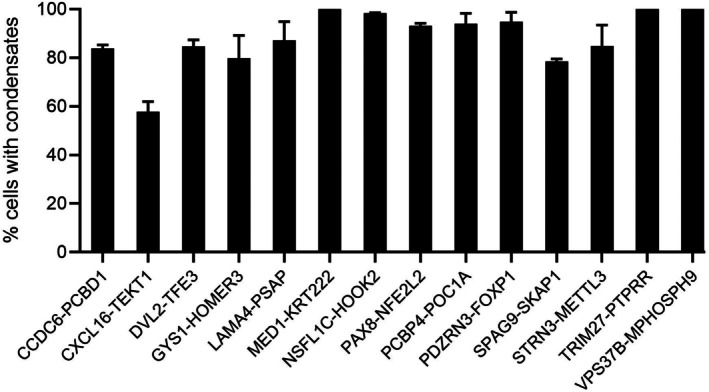
Quantification of condensate formation of fusion proteins. Bar graph shows the percentage of cells exhibiting condensates. Higher percentage suggests higher propensity in forming condensates. Data are presented as mean ± standard deviation from ≥3 independent experiments. For each condition, a minimum of 40 confocal images were acquired per experiment, and approximately 600 cells per construct were analyzed to determine the fraction of condensate‐positive cells.

We quantified the fraction of cells showing condensate formation in Figure 3. We found that all 14 FPs showed a high percentage of condensate formation in cells, though the extent varied among constructs. For example, MED1‐KRT222, NSFL1C‐HOOK2, PCBP4‐POC1A, TRIM27‐PTPRR, and VPS37B‐MPHOSPH9 induced condensates in nearly all cells, indicating high condensate‐forming ability. In contrast, CXCL16‐TEKT1 displayed lower condensate‐positive rate (~60%). Importantly, we did not observe any significant correlation between condensate formation and protein expression levels, as measured by mean mEGFP fluorescence intensity and the fraction of condensate‐positive cells (Figure S2), indicating the observed variation is likely an intrinsic difference in condensate‐forming propensity among CCDs rather than transfection efficiency or protein expression as confounding variables. Overall, the average fraction of condensate‐positive cells across all FPs was ~87% ± 8.3%. The high fraction further suggests that these CCD‐containing FPs have high potency in forming biomolecular condensation.

Further, we have performed additional analyses to quantitatively assess protein expression across all tested constructs, including full‐length FPs, CCD‐only constructs, and CCD‐deleted variants (ΔCCD). Specifically, we measured mean fluorescence intensity at the single‐cell level as a proxy for protein expression in transiently transfected cells. We then examined the relationship between protein expression levels and condensate formation by plotting the fraction of cells exhibiting condensates against the corresponding mean fluorescence intensity for each construct (Figure S2). The lack of any significant correlation between protein expression level and the fraction of condensate‐positive cells across all constructs tested suggests that differences in condensate formation are not primarily driven by variability in protein expression levels.

In addition, our analysis was performed on a single‐cell basis within the transfected population, which minimizes the influence of variations in overall transfection efficiency. Only cells exhibiting detectable fluorescence were included in the analysis, ensuring that comparisons are made among expressing cells. Based on these additional analyses, we believe that the observed differences in condensate formation more likely reflect intrinsic properties of the protein constructs, including the contribution of coiled‐coil domains, rather than differences in expression level or transfection efficiency.

### 2.3 Some coiled‐coil domains alone can drive condensate formation

**FIGURE 4:**
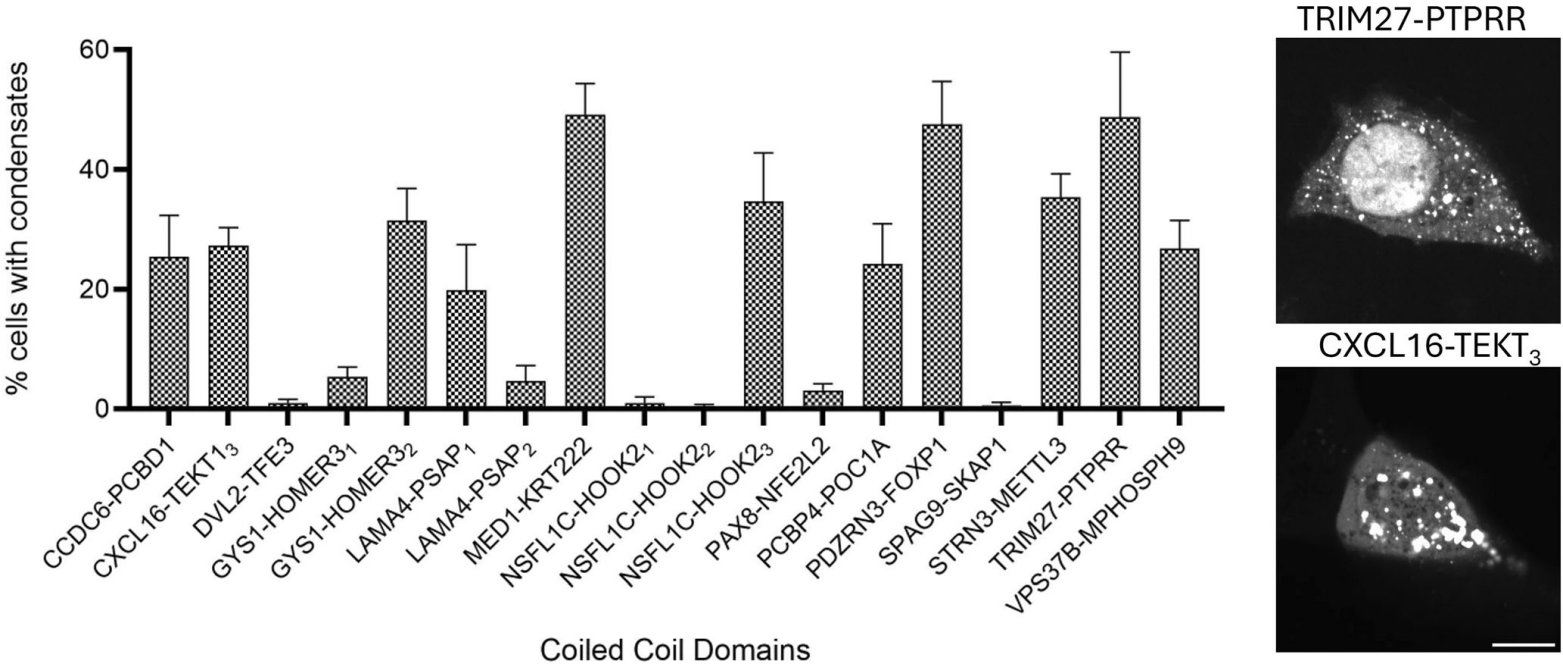
The coiled‐coil domain alone in condensate formation. Bar graph shows the fraction of cells exhibiting condensates. Higher percentage suggests higher potency in forming condensates. Multiple coiled‐coils from the same fusion protein are labeled with a subscript. Data are presented as mean ± standard deviation from ≥3 independent experiments. For each condition, a minimum of 40 confocal images were acquired per experiment, and approximately 600 cells per construct were analyzed to determine the fraction of condensate‐positive cells. Representative images of condensation via coiled‐coil domain alone are shown on the right. Scale bar: 10 μm.

The presence of CCD domains and condensate formation prompted us to determine if CCDs alone can drive condensate assembly. We tested individually all 18 CCDs (Table S2) found in the 14 FPs for their ability to induce condensation in cells. Although these CCDs are only a short fraction of the original full‐length FPs (Table S1), we found about half of them are highly effective in forming condensates on their own (Figures 4 and S3).

Among the strongest condensate drivers, MED1‐KRT222, PDZRN3‐FOXP1, and TRIM27‐PTPRR, we observed ~40% to 50% of cells exhibiting puncta. While the specific fraction is lower than the corresponding full‐length proteins (which is often nearly 100% cells), it is striking that CCDs alone still achieve a significant condensation. Several CCDs, including CCDC6‐PCBD1, PCBP4‐POC1A, CXCL16‐TEKT_3_, STRN3‐METTL3, GYS1‐HOMER3_2_, VPS37B‐MPHOSPH9, and NSFL1C‐HOOK2_2_, showed intermediate activities (~20% to 30%), while LAMA4‐PSAP_1_ formed condensates in ~20% of cells. Here we distinguish multiple CCDs from one FP with a subscript. Additionally, there are CCDs with low‐condensation ability on their own, as we determined with less than 10% condensate‐positive cells. These include DVL2‐TFE3, GYS1‐HOMER3_1_, LAMA4‐PSAP, NSFL1C‐HOOK2_1_, NSFL1C‐HOOK2_2_, PAX8‐NFE2L2, and SPAG9‐SKAP1.

Taken together, these findings demonstrate that CCDs, even when removed from the context of their native protein sequences, can drive condensate formation in cells. The intrinsic self‐associative properties of some CCDs can be sufficient to induce the condensation process. The reduced fraction of condensation in CCD‐alone conditions compared to full‐length FPs suggests that regions outside the CCD can synergize with the CCD to promote condensate formation, possibly by providing additional interaction surfaces within the full‐length protein (Figure S4).

We performed additional experiments in which mEGFP was replaced with mNeonGreen, a structurally distinct monomeric fluorescent protein with no known tendency to self‐associate (Barkley et al., 2024). Across all 18 CCD constructs, all mNeonGreen‐tagged CCD constructs were cloned into the same pcDNA3.1 backbone vector used for the original mEGFP‐tagged constructs, ensuring that any differences in condensate behavior could be attributed specifically to the fluorescent tag rather than to other construct‐level variables. Condensate formation was assessed by fluorescence microscopy under identical imaging conditions, and the fraction of condensate‐positive cells was quantified for each construct. As shown in Figure S5, we observed no statistically significant differences in condensate formation propensity between the mEGFP‐tagged and mNeonGreen‐tagged versions of any of the 18 CCD constructs tested. Specifically, the rank ordering of condensate formation propensity across constructs was preserved between the two tagging conditions, and the classification of CCDs into high‐ and low‐condensate‐forming groups was entirely consistent regardless of which fluorescent tag was used. These results strongly rule out the possibility that the condensate formation behavior observed in our study is driven or artifactually augmented by properties of the mEGFP tag.

### 2.4 A “triad‐extension” model for higher‐order assembly of coiled‐coil domains

We first tested whether condensate‐forming propensity could be explained by simple sequence‐level features describing e/g‐position electrostatics and a/d‐position hydrophobic core properties. These features included the overall fraction of charged residues at e/g positions, the fractions of positively and negatively charged residues at e/g positions, the net sequence charge of each CCD, and the mean Kyte‐Doolittle hydropathy and hydrophobic residue fraction at a/d core positions. All of these descriptors showed only weak correlations with condensate‐forming propensity, including e/g charged fraction (Spearman's *ρ* = −0.295, *p* = 0.234), e/g positive‐charge fraction (*ρ* = −0.344, *p* = 0.163), e/g negative‐charge fraction (*ρ* = 0.040, *p* = 0.874), net sequence charge (*ρ* = 0.279, *p* = 0.262), mean Kyte‐Doolittle hydropathy at a/d positions (*ρ* = −0.205, *p* = 0.414), and hydrophobic residue fraction at a/d positions (*ρ* = −0.144, *p* = 0.569). Thus, bulk sequence composition alone was insufficient to explain the observed differences among CCDs. We therefore developed a triad‐extension model that explicitly evaluates how the spatial arrangement and pairwise compatibility of charged residues at e/g positions may promote or prevent multichain extension.

**FIGURE 5:**
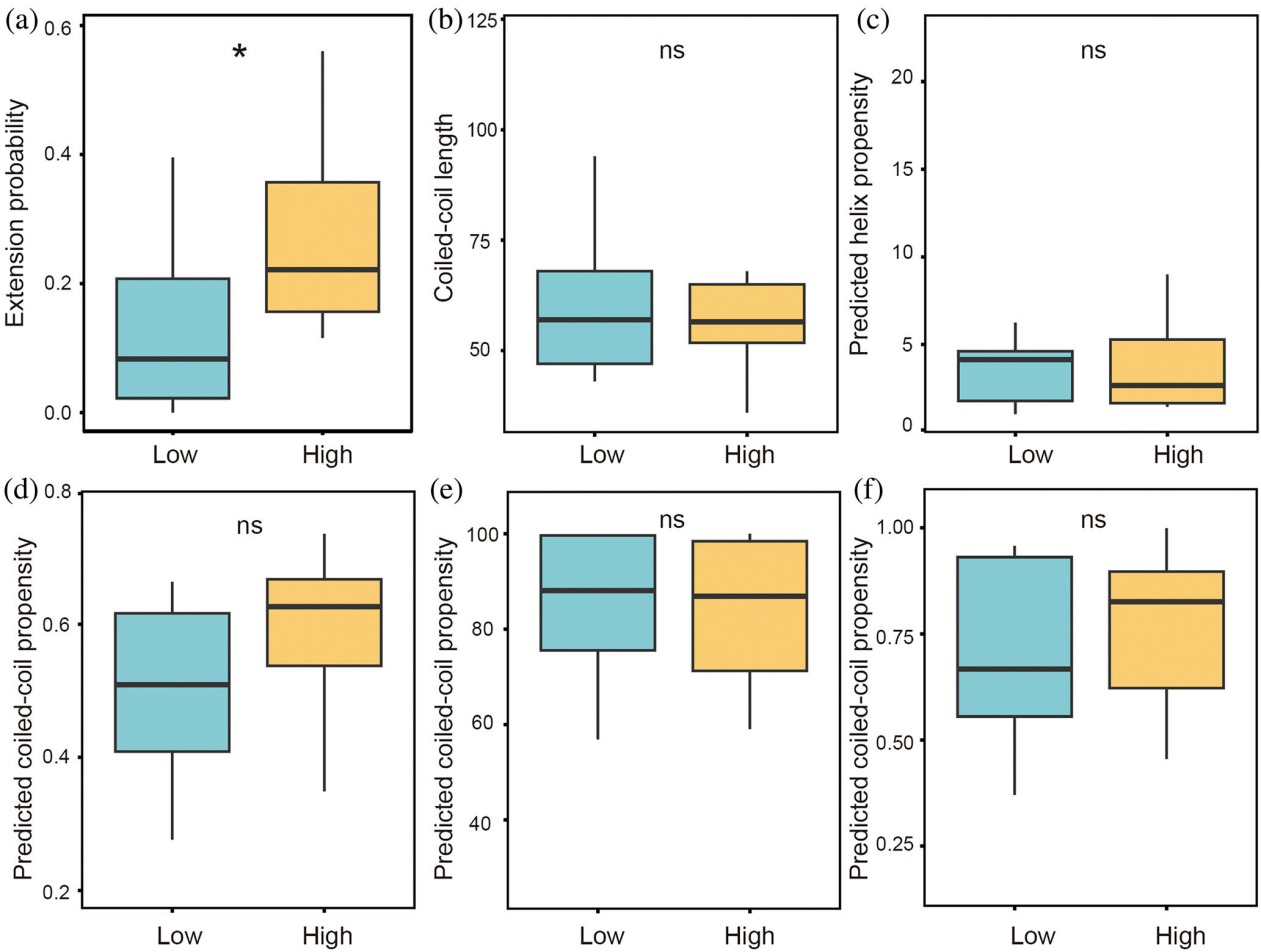
Comparison of sequence‐based features and triad‐model‐guided mutational validation of coiled‐coil condensation propensity. Box plots show (a) triad‐model extension probability, (b) coiled‐coil length, (c) Agadir‐predicted helix propensity, and coiled‐coil propensity predicted by (d) DeepCoil2, (e) MARCOIL, and (f) PCOILS for coiled‐coils with low or high condensation capability. Statistical significance was assessed using the Wilcoxon rank‐sum test; *p < 0.05; ns, not significant.

Although Figure 4 demonstrates that coiled‐coil domains alone can be sufficient to induce condensation, the molecular basis by which they assemble beyond their classical oligomeric states (i.e., mostly dimers or trimers as listed in Table S3) into higher‐order micrometer‐scale structures remains incompletely understood. Previous work has shown that coiled‐coil interfaces can adopt axially shifted, sliding‐related binding configurations, indicating that dimer formation is not necessarily restricted to perfectly end‐aligned states (Gomez et al., 2019). Consistent with the idea that such non‐classical encounter geometries may support continued assembly growth, a recent modeling study further suggested that single coiled‐coil domains can undergo higher‐order recruitment through offset interactions, potentially leading to extended fiber‐like assemblies (Ramirez et al., 2024). Here, our model does not describe dynamic sliding of a preformed dimer, but instead enumerates possible stochastic helix–helix encounter configurations, including axially offset states, and evaluates their compatibility with third‐chain recruitment. Indeed, this possibility became particularly relevant because conventional coiled‐coil characterization methods showed no significant difference between the condensate‐promoting and condensate‐deficient coiled‐coils (Figure 5b–f). Properties such as coiled‐coil length, helical propensity measured by Agadir algorithm (Figure 5c), coiled‐coil formation probabilities predicted by DeepCoil2 (Figure 5d), Marcoil (Figure 5e), or Pcoils (Figure 5f) all fail to distinguish between the two different groups of coiled‐coils.

To understand the underlying driving force for condensation mediated by coiled‐coil domains, we first calculated the stability of dimers in different offset positions (i.e., sliding one or more heptads along the dimer central axis). This offsetting is not typically considered in other theoretical framework to model coiled‐coil dimer interaction. We found that, as expected, dimers with perfect end‐to‐end alignment formed the most stable complexes, owing to the larger number of favorable residue interactions. However, several axially shifted offset configurations also exhibited relatively favorable interaction energies, indicating that stable non‐end‐aligned encounters are also possible within the model framework (Figure S6). We reason that these stable offset dimers have available binding sites at their two ends to accommodate a third chain. If the resulting triad is energetically favorable, the triad will continue to grow and the process repeats for further chain extension (Figure S7). We therefore calculate the extension probability of a triad based on the electrostatic interactions between heptad “e”/“g” positions. Applying this mechanistically defined triad‐extension model to the analyzed sequences, we found that condensate‐promoting proteins exhibited significantly higher extension probabilities than condensate‐deficient proteins (Figures 5a and S9), whereas coiled‐coil length (Figure 5b) and all other coiled‐coil predictors did not differ significantly between the two groups (Figure 5c–f).

**FIGURE 6:**
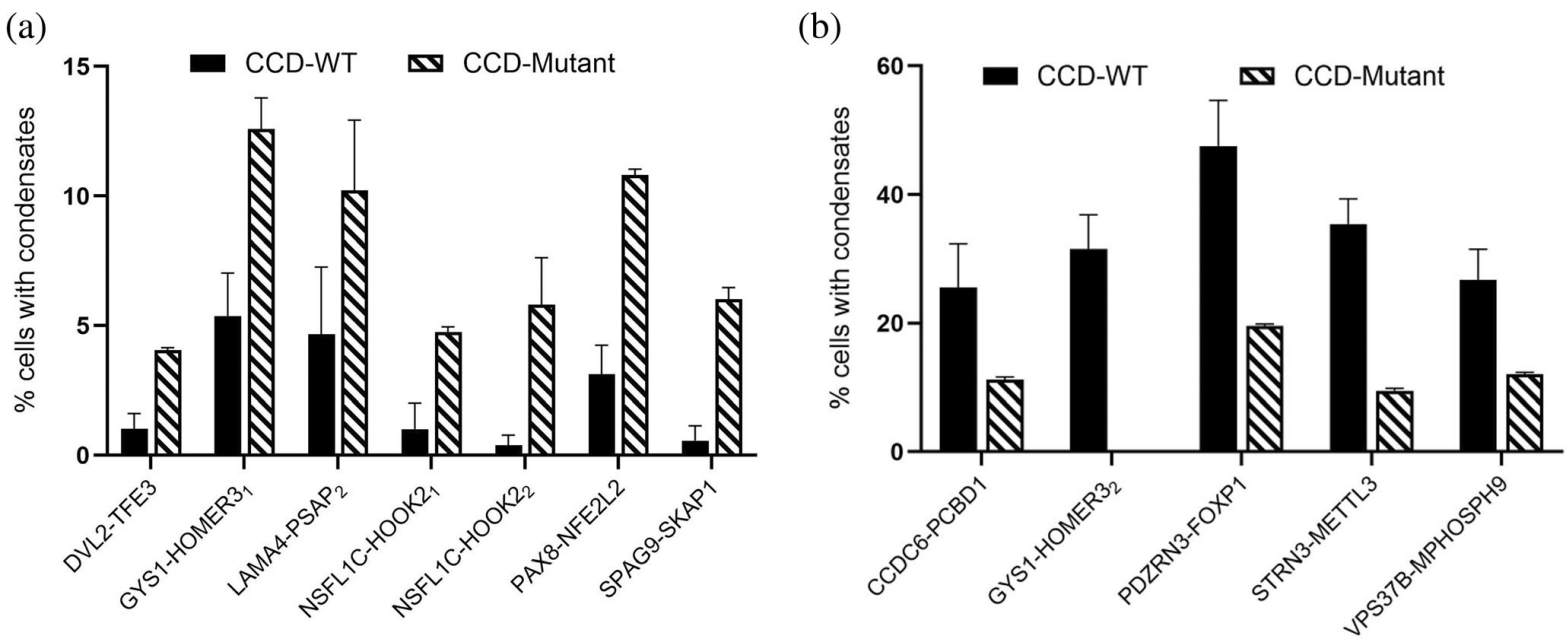
Effect of charge mutations on condensate formation in fusion proteins with low‐ and high‐condensation coiled‐coil domains (CCD). (a) CCD mutations designed to increase coiled‐coil extension probability in fusion proteins enhance LLPS. (b) CCD mutations designed to decrease coiled‐coil extension probability in fusion proteins reduce the percentage of cells with condensates. Data are presented as mean ± standard deviation from ≥3 independent experiments. For each condition, a minimum of 40 confocal images were acquired per experiment, and approximately 600 cells per construct were analyzed to determine the fraction of condensate‐positive cells.

To further test the predictive power of the triad‐extension model, we introduced targeted substitutions at 2–5 e/g positions in selected coiled‐coil domains to alter charge properties and thereby tune the predicted extension score. In selected low‐condensation CCDs, mutations predicted to increase the extension score increased the experimentally measured percentage of cells with condensates (Figure 6a, Table S4). In the high‐condensation group, we focused on five coiled‐coils in which the triad‐extension score showed relatively good agreement with the experimentally measured condensation propensity, and mutations predicted to decrease the extension score reduced the percentage of cells with condensates (Figure 6b, Table S4). Together, these results show that modifying charge properties at e/g positions can tune the triad‐extension score and shift condensation behavior in the expected direction.

These mutational results further support e/g‐position electrostatics as an important determinant of CCD‐mediated condensation. We therefore considered how this electrostatics‐focused mechanism relates to prior models of offset coiled‐coil assembly. This result is consistent with a seminal study by Ramirez et al. (2024) which showed CCDs can be held together with offset interactions. In that model, perfect hydrophobic interactions at a/d positions in the coiled‐coil sequences can propagate into extended fiber‐like assemblies. In contrast, the present study examines naturally occurring coiled‐coil sequences, many of which contain non‐ideal residues at a/d core positions which may weaken the stability and continuity of offset hydrophobic packing. These differences suggest that offset‐binding geometries can give rise to different mesoscale outcomes, from dynamic condensates to more stable fiber‐like assemblies, depending on sequence context and interaction stability.

It is important to note that the extension probability derived here should be interpreted as an intrinsic property of the isolated coiled‐coil segment. In the context of a full‐length protein, flanking regions may alter the accessibility, frequency, and geometrical feasibility of these interactions, and therefore influence whether the predicted alignments can be realized in practice. Thus, the model output is not intended as a complete prediction of coiled‐coil behavior in the full‐length protein context.

### 2.5 The role of coiled‐coil domain in condensate formation compared with full‐length fusion proteins

**FIGURE 7:**
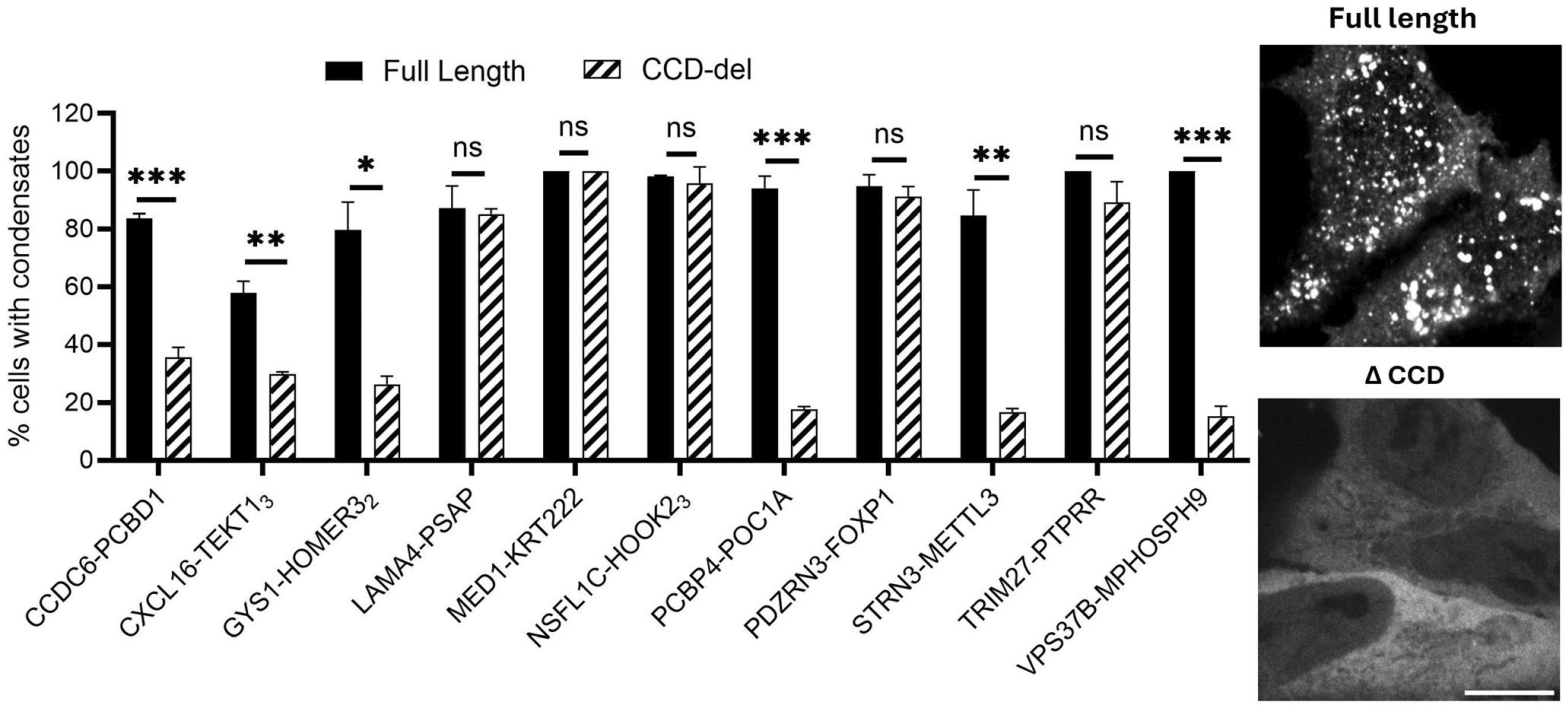
The comparison between full‐length fusion proteins and coiled‐coil domain (CCD) deletion mutants in condensate formation. Quantification of the fraction of cells with condensates is shown. Full‐length proteins are shown as black bars and CCD‐deletion mutants (ΔCCD) are shown as hatched bars. Data are presented as mean ± standard deviation from ≥3 independent experiments. For each condition, a minimum of 40 confocal images were acquired per experiment, and approximately 600 cells per construct were analyzed to determine the fraction of condensate‐positive cells. Representative fluorescence microscopy images (right) illustrate condensates in full‐length PCBP4‐POC1A protein and reduced condensation in the corresponding ΔCCD mutants. Statistical significance was determined by unpaired *t*‐test (ns = not significant; *p < 0.05; **p < 0.01; ***p < 0.001). Scale bar: 10 μm.

After demonstrating the condensate‐forming ability of CCDs on their own, such as the 11 CCDs that exhibit high condensate activity shown in Figures 4 and S3, we next investigated whether their presence was the sole determining factor in FP condensate assembly. To achieve this, we compared the full‐length FPs with the CCD‐deleted mutants (ΔCCD) and quantify the fraction of cells with condensate formation in Figure 7. Full‐length FPs consistently formed condensates in a majority of cells, typically ranging from ~60% to nearly 100%. In contrast, deletion of the CCD caused a reduction in condensate formation in many FPs. We observed three proteins (PCBP4‐POC1A, STRN3‐METTL3, and VPS37B‐MPHOSPH9) condensate formation was almost completely abolished following CCD deletion (*p* < 0.001). While full‐length proteins assembled condensates in ~70% to 90% of cells, the fraction of cells with condensates in those ΔCCD proteins fell below 15%, demonstrating that the CCD is a critical determining factor for condensation in these proteins. Similarly, another subset of proteins (CCDC6‐PCBD1, CXCL16‐TEKT1_3_, and GYS1‐HOMER3_2_) showed a markedly reduced ability to form condensates in the absence of the CCD. In these proteins, full‐length proteins formed puncta in ~60% to 80% of cells, while condensation of ΔCCD proteins is significantly reduced (~20% to 30%; *p* < 0.01). Interestingly, five CCDs (from LAMA4‐PSAP, MED1‐KRT222, NSFL1C‐HOOK2_3_, PDZRN3‐FOXP1, and TRIM27‐PTPRR) did not show a significant effect in reducing the overall protein condensation. The comparison suggests that while CCDs are important drivers in condensation of many FPs, additional factors (such as RNA‐binding motifs or low‐complexity regions) may also contribute to condensate formation.

This work identifies the contribution of specific coiled‐coil domains in promoting condensation of FPs. Previous studies suggested that FPs can employ IDRs to drive condensate assembly (Farag et al., 2022; Fonin et al., 2022; Lee et al., 2022; Tripathi et al., 2023). Our observation shows that additional mechanisms can promote condensate formation. Unlike the well‐known mechanism of IDRs driving protein condensation, the condensate assembly in this study is driven by the self‐association between well‐defined structured domains. Furthermore, we show that certain coiled‐coil domains alone, without the surrounding protein context, can be sufficient in promoting condensate assembly. The methodology developed in this study can be readily applicable to study the contribution of other domains in protein condensation. For example, when incorporating algorithms to identify other structured domains or protein families, their respective contribution to condensate formation can be systematically analyzed. Our approach of quantitative imaging and domain deletion assays can work in various systems to pinpoint specific roles of the domains of interest. The naturally occurring sequences identified in this work could be further adopted and engineered in other artificial condensation systems.

## 3 MATERIALS AND METHODS

### 3.1 Fusion protein data curation and selection criteria

FP entries (*n* = 48,589) were initially compiled from publicly available databases The Cancer Genome Atlas (TCGA) (Tomczak et al., 2015) and ChiTaRS (Souza et al., 2025). Those containing transmembrane domains were excluded using TMHMM algorithm, resulting in a dataset of 34,631 entries. Coiled‐coil domain (CCD) prediction was performed using DeepCoil2, a deep‐learning‐based tool for identifying coiled‐coil regions in proteins (Ludwiczak et al., 2019). Selection criteria on CCD length and probability score were applied to ensure high‐confidence CCD identification. One thousand and one‐hundred and sixty‐five proteins were found to contain predicted CCDs. After removing redundant splicing isoforms, 222 unique FPs remained. From these, a final subset of 18 CCDs from 14 FPs was selected using the following criteria: total protein length <1000 amino acids, CCD probability score ≥0.5, CCD length ≥35 amino acids, and presence of canonical heptad pattern. This multi‐tiered selection strategy enabled the identification of a tractable set of FPs with structured CCDs suitable for downstream characterization.

### 3.2 Construct design and cloning of fusion proteins

All constructs were cloned into an identical backbone vector (pcDNA3.1) with an mEGFP reporter gene tagged at the N‐terminus. Briefly, fusion genes with an mEGFP tag were assembled following the Takara In‐Fusion Snap Assembly protocol (cat. 060822). The full‐length FP plasmids were then used to create CCD‐only and CCD‐deleted constructs. CCD‐only constructs were created by amplifying the CCD region using CCD‐specific primers. CCD‐deleted constructs were created using Q5® Site‐Directed Mutagenesis Kit (New England Biolabs, Inc. USA). The resulting product was re‐circularized using a Kinase, Ligase, Dpnl reaction (NEB #M0554). The plasmids were then transformed into NEB® 5‐alpha Competent *Escherichia coli* cells (New England Biolabs, Inc. USA) following the NEB High Efficiency Transformation protocol (NEB C2987H). Positive colonies were selected on an Ampicillin containing luria‐bertani medium agar plates and then plasmids were purified using the ZymoPURE Plasmid Miniprep Kit (Irvine, CA, USA). Positive clones were confirmed by Sanger sequencing of the plasmids was conducted by ACGT, Inc. (Wheeling, IL, USA). All mutational fragments were synthesized using the integrated DNA technologies (IDT) gBlock service, and the fragments were assembled into an mEGFP‐tagged pcDNA3.1 vector backbone following the Takara In‐Fusion Snap Assembly protocol.

### 3.3 Cell culture

U2OS and HeLa cells were maintained in humidified incubators with 5% CO_2_ at 37°C. Cells were cultured in dulbecco's modified eagle medium (Corning #10‐013‐CV) supplemented with 10% (v/v) heat‐inactivated fetal bovine serum, and penicillin streptomycin. Mycoplasma testing was routinely performed using a MycoAlertTM Mycoplasma Detection Kit (Lonza # LT07‐418).

### 3.4 Fluorescence microscopy and image analysis

Twenty thousand cells were plated on each well of an 8‐well plate (Cellvis) and allowed to incubate at 37°C for 24 h before transfection. Cells were transfected according to the manufacturer's protocol. Two hundred and fifty nanograms of plasmid was combined with 25 μL of opti‐MEM and 0.75 μL of transfection reagent (Mirus TransIt‐LT1 transfection reagent) and set to incubate at room temperature for 30 min before being added to one well of an 8‐well plate. Cells were imaged 24 h after transfection using confocal microscopy. For each condition, 40 images were acquired per experiment, and experiments were independently repeated three times. Condensate‐positive cells were identified as those exhibiting at least three discrete fluorescent puncta above background fluorescence levels. A minimum of 600 cells per construct were counted across all replicates to determine the fraction of condensate‐positive cells. Imaging was done with a Nikon Ti2 microscope with a CSU‐W1 spinning disk confocal scanning unit using a 100x/1.4 Plan Apo VC objective. Images were analyzed using ImageJ software. Further, to control for potential confounding effects of differential protein expression and transfection efficiency, mean fluorescence intensity was measured for each mEGFP‐positive cell using ImageJ software. For each construct, a minimum of 600 mEGFP‐positive cells were analyzed across three independent experiments. The fraction of condensate‐positive cells was then plotted against mean fluorescence intensity per construct to assess whether protein expression level correlated with liquid‐liquid phase separation (LLPS) propensity.

To assess whether the mEGFP fluorescent tag influences condensate formation propensity, all 18 CCD constructs were additionally cloned into the pcDNA3.1 backbone with mEGFP replaced by mNeonGreen using the same Takara In‐Fusion Snap Assembly protocol. Condensate formation was assessed by fluorescence microscopy and quantified as described in Section 3.4. The fraction of condensate‐positive cells was compared between mEGFP‐ and mNeonGreen‐tagged versions of each construct to determine whether tag identity influenced condensate formation behavior.

### 3.5 Analysis of coiled‐coil domain features with existing algorithms

To evaluate sequence‐based structural propensities of coiled‐coil domains, we performed secondary‐structure and coiled‐coil predictions using multiple established algorithms. Helical propensities were computed using the Agadir algorithm, which estimates α‐helical stability based on amino acid composition and sequence context (Lacroix et al., 1998). Calculations were performed under physiological conditions, assuming non‐capped termini, neutral pH (7.0), a temperature of 310 K, and an ionic strength of 0.15M. The probability of coiled‐coil formation propensity values were further predicted using DeepCoil2 (Ludwiczak et al., 2019), Marcoil (Delorenzi & Speed, 2002), and PCOILS (Gruber et al., 2006), all of which identify heptad repeat patterns and interhelical pairing tendencies. These sequence‐based propensity values were then compared between the high and low‐condensation groups to assess whether coiled‐coil or helical propensities could distinguish their differential condensation behavior.

### 3.6 Identification of coiled‐coil segments and heptad mapping

DeepCoil2 was used to annotate coiled‐coil regions from primary sequences (Ludwiczak et al., 2019), and residue‐level prediction scores were used to identify coiled‐coil segments (Figure 1). Subsequent computational analyses on residue interactions were based on the assigned heptad positions, which allowed the model to evaluate all possible interactions within a given triad geometry directly. To verify that these sequences were compatible with canonical heptad packing (Lupas et al., 2017; Madaj et al., 2025), we cross‐validated all coiled‐coils using AlphaFold2 (Jumper et al., 2021) and analyzed the predicted structures with Socket2 (Kumar & Woolfson, 2021) to assign coiled‐coil registers (Figure S8).

### 3.7 Modeling coiled‐coil dimer interactions and triad‐extension

Previous work has shown that coiled coils capable of forming axially overlapping interactions can self‐assemble into higher‐order networks and undergo liquid–liquid phase separation in vitro (Ramsak et al., 2023). We therefore model how two α‐helices, upon random encounter in solution, can form such overlap‐competent contacts that propagate into larger assemblies and ultimately promote protein condensation. For each construct, possible encounter geometries were enumerated by considering both parallel (P) and antiparallel (AP) orientations and by axially sliding one chain relative to the other across all offsets that yield at least one full heptad overlap. Pairing rules followed canonical coiled‐coil geometry. In the P orientation, residues at “e” positions on one helix contact “g” positions on the other. In the AP orientation, the electrostatic contacts occur between like registers (“e”—“e” and “g”—“g”), rather than “e”—“g” swapping (Figure S6).

For each alignment and for each interface (i.e., a specific pair of chains at a given orientation/offset), we evaluated electrostatic complementarity at the charged heptad positions using fixed residue charges: lysine +1, arginine +1, histidine +0.5, aspartate −1, glutamate −1 (Joseph et al., 2021); all other residues 0. Opposite‐signed pairs contributed +1 (or +0.5 when histidine was involved), like‐signed pairs −1 (or −0.5 with histidine), and pairs with any uncharged residue contributed 0.

For each offset dimer, say chain A and chain B, we assessed its ability to recruit an additional chain by pairing it with a third chain (C) at every possible binding position to form triads (Figure S7). For each triad, the two interaction scores (i.e., between A and B chains; and between B and C chains) were computed and the stability of the triad was determined by the smaller value of the two values. For each interaction interface, the electrostatic score was calculated as the number of favorable oppositely charged pairings minus the number of unfavorable like‐charged pairings. A triad was considered stable only when both interfaces had a positive score, equivalent to requiring the smaller of the two interface scores to be greater than 0, corresponding to net favorable electrostatic complementarity at both interfaces. We defined the extension probability of a given protein sequence as the fraction of stable triads among all possible A‐B‐C triad configurations. In other words, the higher the fraction of stable triads, the more likely the protein will form triads to recruit additional chains, thereby nucleating multichain growth and facilitating condensate formation. For each sequence, electrostatic interface scores were computed for all orientations and axial offsets, and the proportion of stable triads was used to compute the extension probability.

### 3.8 Statistical analysis

Scores derived from the triad‐extension model, sequence length, and predictions from Agadir, DeepCoil2, Marcoil, and PCOILS were statistically compared between high‐ and low‐condensation coiled‐coil groups using the Wilcoxon rank‐sum test implemented in R (version 4.5.0). The triad‐extension simulations were also performed in R (version 4.5.0) using custom scripts. GraphPad Prism (GraphPad Software, La Jolla, CA) and Microsoft Excel were used to analyze the experimental data. One‐way analysis of variance (ANOVA) test was used to determine statistical significance (*p ≤ 0.05, **p ≤ 0.01, ***p ≤ 0.001). All data are indicated as mean ± SD (standard deviation) for *n* ≥ 3.

## AUTHOR CONTRIBUTIONS

**Carter Humphrey:** Methodology; formal analysis; data curation; visualization. **Juan Guan:** Conceptualization; writing – review and editing; writing – original draft; funding acquisition; investigation; resources; project administration; supervision. **Lu Liu:** Conceptualization; software; visualization; validation; methodology; writing – review and editing; writing – original draft; formal analysis; investigation. **Kyle Scheller:** Methodology; investigation; formal analysis; data curation; writing – review and editing. **Om Prakash Narayan:** Conceptualization; investigation; writing – original draft; methodology; validation; writing – review and editing; formal analysis; data curation; visualization. **Jiawei Dong:** Methodology; formal analysis; data curation; visualization.

## CONFLICT OF INTEREST STATEMENT

The authors declare no conflict of interests.

## Supporting information


**Data S1.** Supporting Information.

## Data Availability

All data needed to evaluate the conclusions in the paper are present in the paper and/or the Supporting Information S1.
