# Supplementary material for: Fusion protein condensate formation via coiled‐coil domains
Source: Protein Sci. 2026 Jun 12;35(7):e70674. doi: 10.1002/pro.70674 (PMC13261776; doi:10.1002/pro.70674)
Supplement: Supplementary file 1 — Data S1. Supporting Information. [file PRO-35-e70674-s001.pdf]

Supplementary Information For:

Fusion Protein Condensate Formation via Coiled-Coil Domains

Om Prakash Narayan[1,#] Lu Liu[1,#] Kyle Scheller[2,3,#] Carter Humphrey[4], Jiawei Dong[1], Juan Guan[1]

[1]Division of Chemical Biology and Medicinal Chemistry, College of Pharmacy, University of Texas at Austin, Austin, TX 78712, USA

[2]Department of Molecular Genetics and Microbiology, University of Florida College of Medicine, Gainesville, FL 32610, USA

[3]University of Florida Health Cancer Center, Gainesville, FL 32610, USA

[4]Biotechnology Department, Austin Community College, Austin, TX 78752, USA

# equal contribution

Correspondence

Juan Guan, The University of Texas at Austin, 2409 University Avenue, Austin, TX 78712, USA.

Email: juanguan@utexas.edu

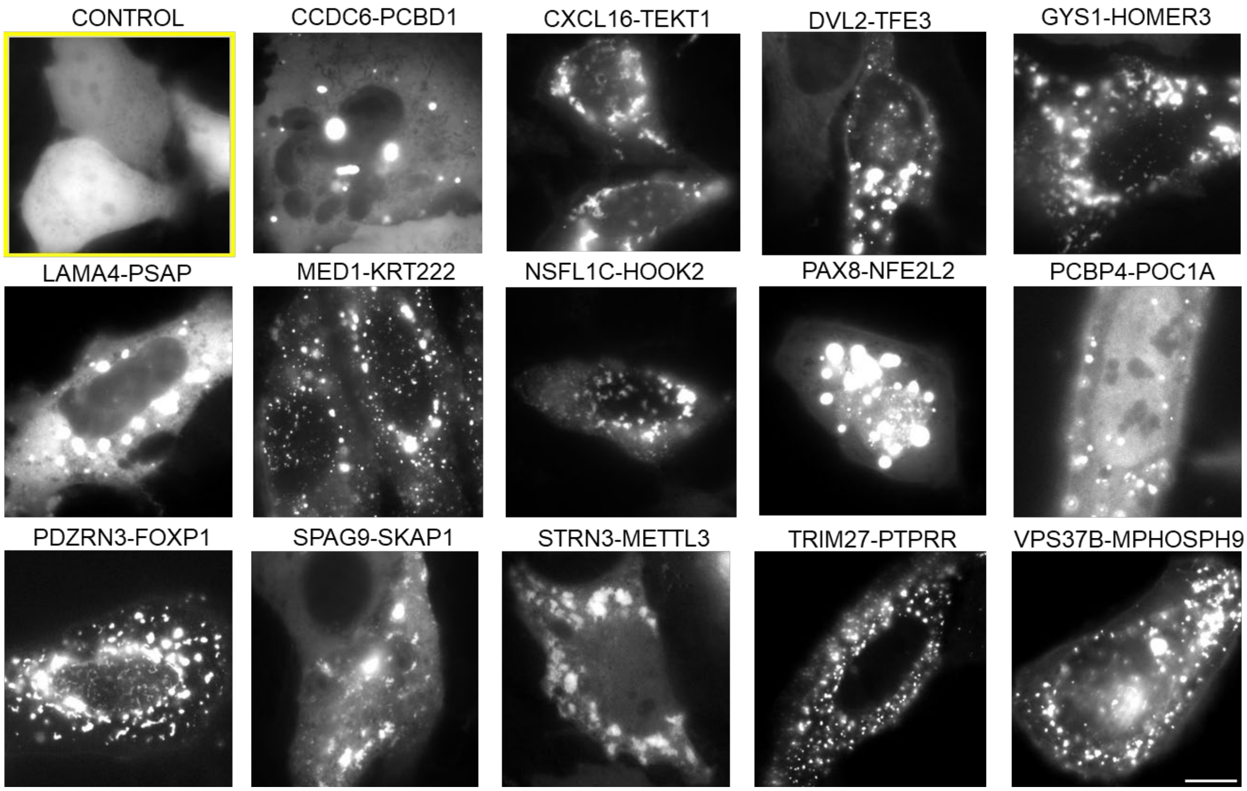

Supplemental Figure S1. CCD-containing fusion proteins form condensates in HeLa cells, consistent with results in U2OS cells. Bright puncta indicate condensate formation. Control group (highlighted yellow box) expressing mEGFP alone showed diffuse distribution without condensates. Scale bar: 10 μm.

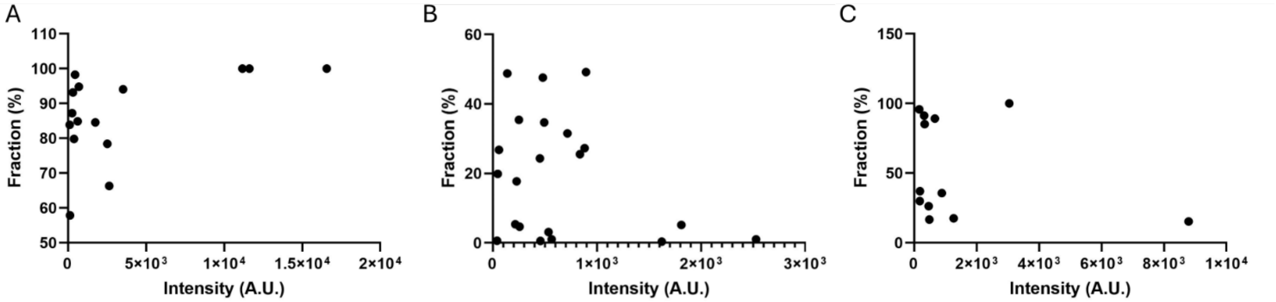

Supplemental Figure S2. Condensate formation is independent of protein expression level. (A–C) Scatter plots showing the fraction of cells exhibiting condensates as a function of mean fluorescence intensity (proxy for protein expression) for (A) full-length fusion proteins, (B) CCD-only constructs, and (C) CCD-deleted (ΔCCD) variants. Each data point represents an individual protein, with values averaged across multiple cells. No significant correlation is observed between protein expression level and the fraction of condensate-positive cells in any group, indicating that differences in condensate formation are not driven by expression variability. Data are presented as mean ± SD from ≥3 independent experiments, with ~600 cells analyzed per protein per experiment. Statistical analysis was performed using linear regression, and no significant correlation was detected ($p > 0.05$).

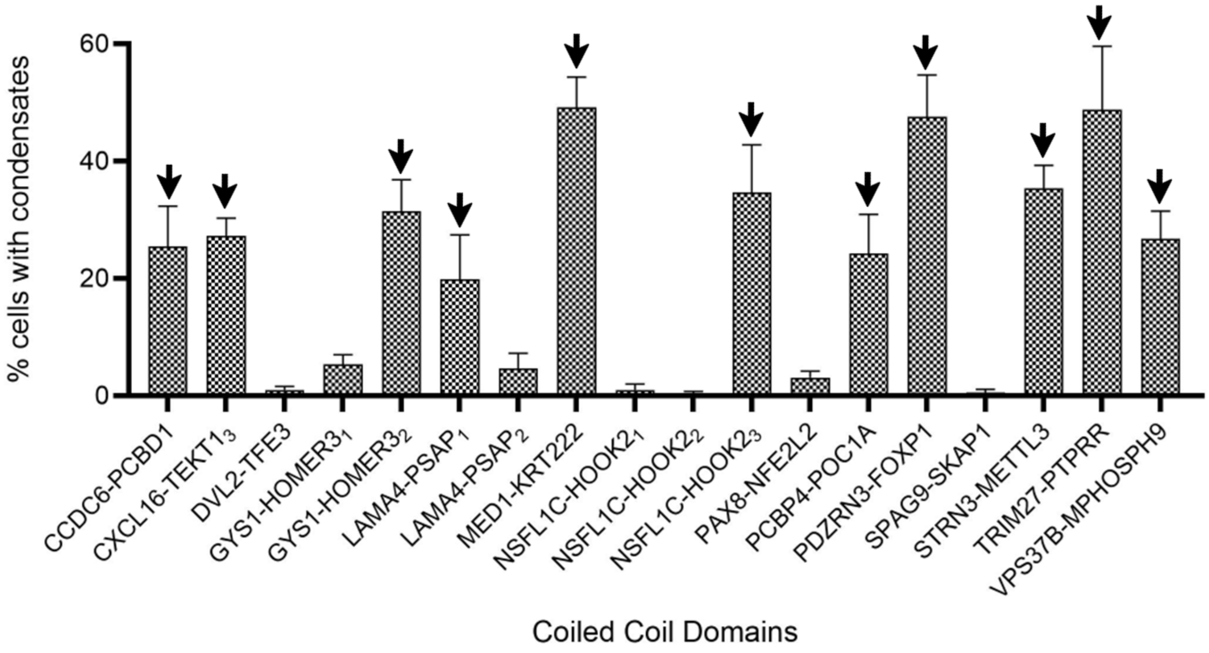

Supplemental Figure S3. The coiled-coil domains in condensate formation. Bar graph shows the fraction of cells exhibiting condensates. The black arrowheads highlight all the proteins in the "high" group of condensate formation capability. The coiled-coil domains in condensate formation. The bar graph shown here is identical to that presented in Figure 4 of the main text, and all data are derived from the same experiments described therein, with 40 images acquired per condition per experiment and a minimum of 600 cells counted per construct across three independent replicates. This supplemental figure is provided solely to annotate the data with black arrowheads, which highlight all coiled-coil domains belonging to the 'high' condensate formation capability group, as referenced in the triad-extension model analysis (Section 2.4). Data are presented as mean ± SD.

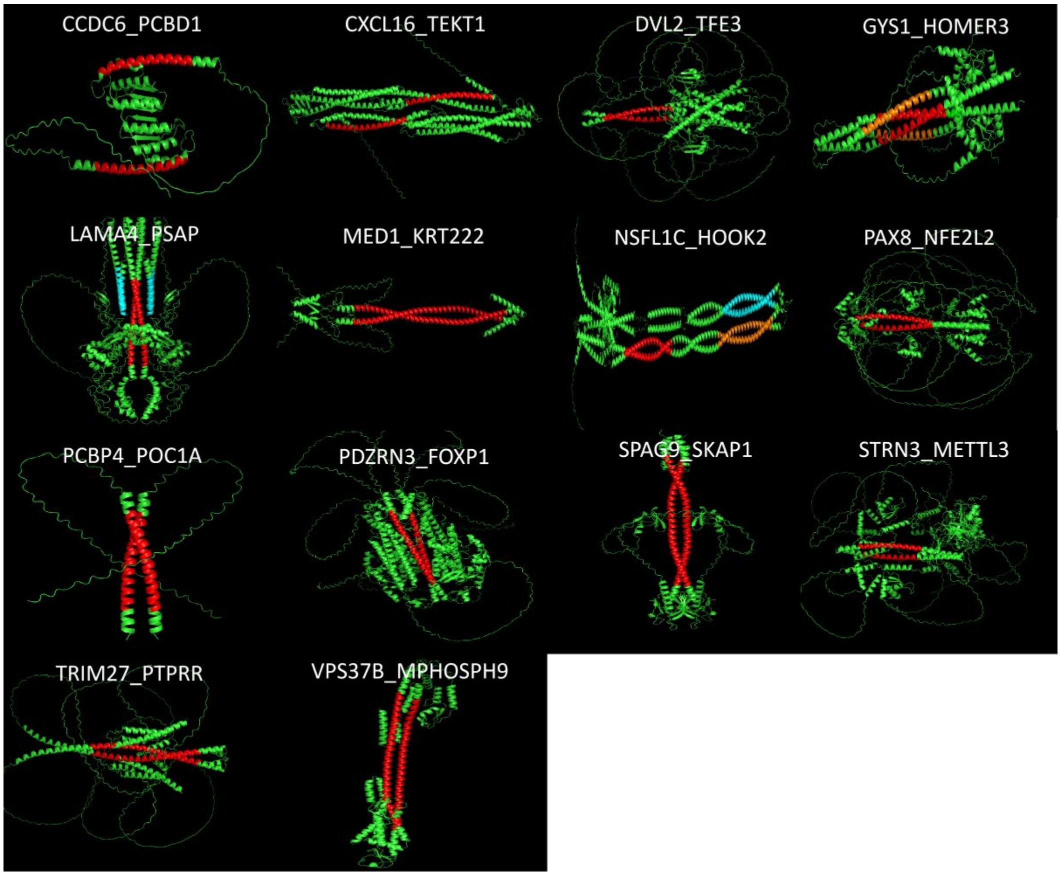

Supplemental Figure S4. AlphaFold-based full-length multimer models revealed that CCDs occupy diverse structural contexts across fusion proteins, ranging from exposed elongated segments to elements embedded within larger helical or folded assemblies, consistent with context-dependent enhancement of condensate formation by non-CCD regions. Backbone context is shown in green.

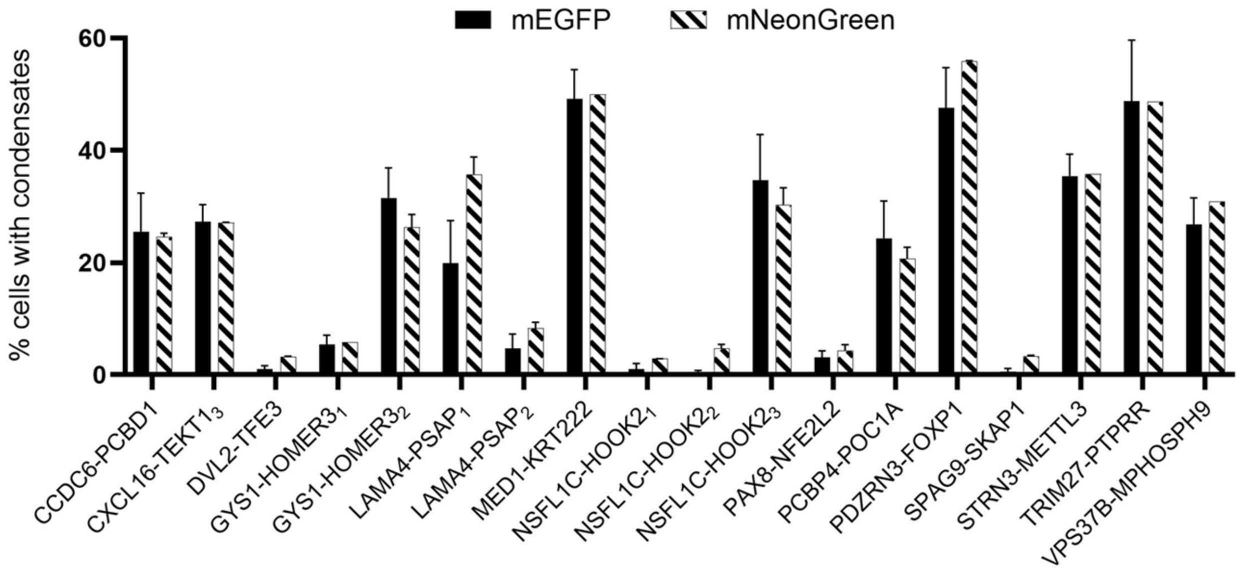

Supplemental Figure S5. Comparison of condensate formation using different fluorescent tags across CCD constructs. Bar graph showing the fraction of cells exhibiting condensates for 18 CCD constructs tagged with mEGFP and mNeonGreen. Each pair of bars represents the same CCD. Overall, the condensate-forming propensity and relative trends across constructs are largely consistent between mEGFP- and mNeonGreen-tagged proteins, with no systematic increase or decrease attributable to a specific tag. Data are presented as mean ± SD from ≥3 independent experiments, with ~600 cells analyzed per construct per experiment. Statistical comparisons between mEGFP and mNeonGreen conditions for each construct show no significant differences ($p > 0.05$, unpaired two-tailed t-test), indicating that condensate formation is not significantly influenced by the choice of fluorescent tag.

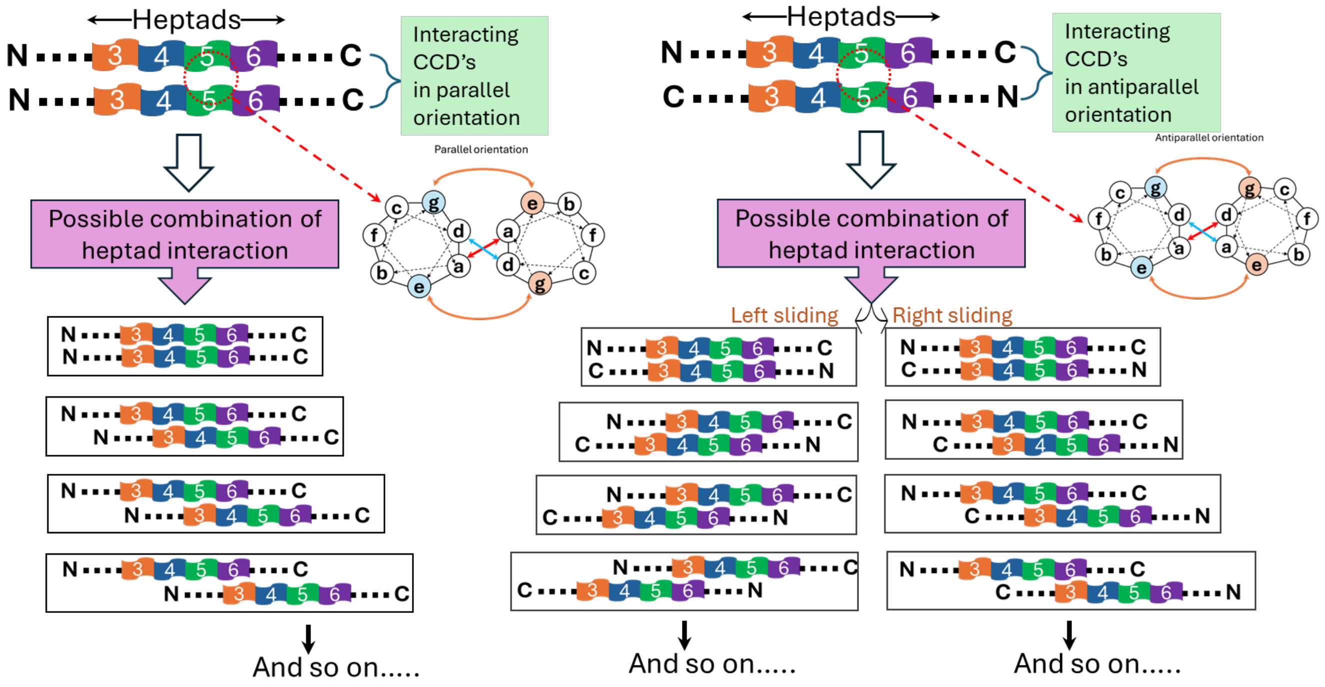

Supplemental Figure S6. A schematic of offset coiled-coil pairing and interaction pattern. Heptad labels (*a*–*g*) were determined to identify hydrophobic (a/d) and electrostatic (e/g) sites. All parallel (P) and antiparallel (AP) dimer geometries were generated by sliding one heptad over the other across all offsets with ≥1 heptad overlap (P: e↔g; AP: e↔e, g↔g).

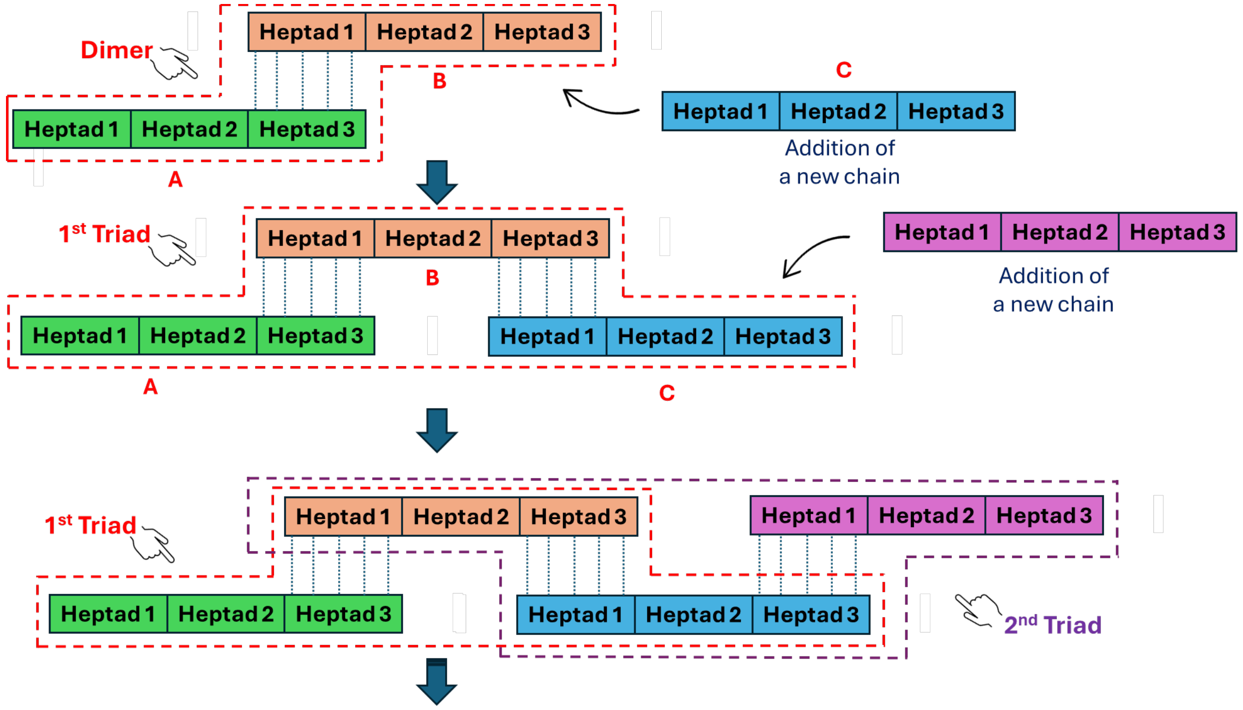

Supplemental Figure S7. Formation of stable dimer and further progression of stable triad (chain A, B and C) based on heptad pairing. Schematic illustrates an example of a coiled-coil domain with three heptads.

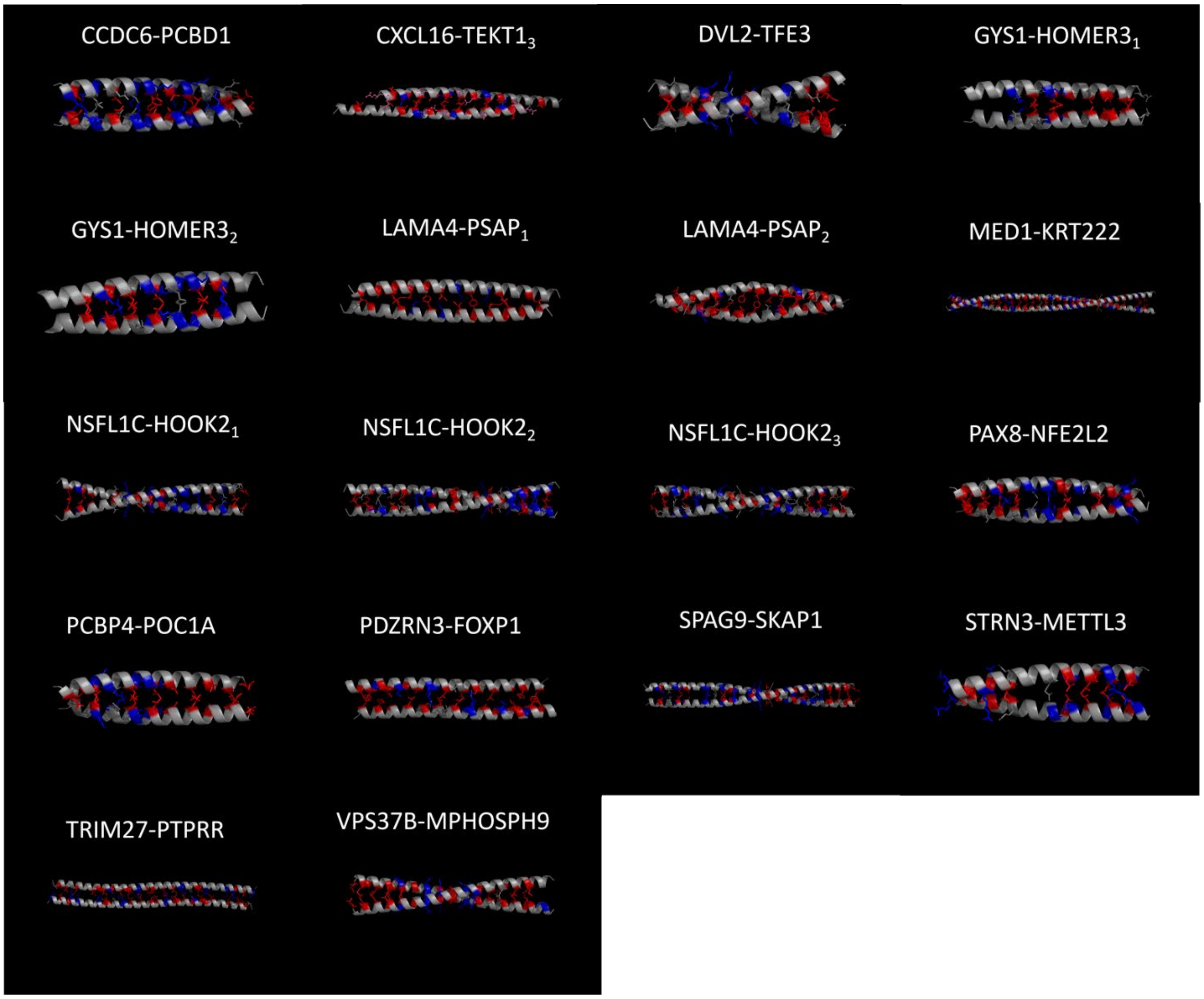

Supplemental Figure S8. AlphaFold2 predicted coiled-coil models and heptad register assignment. Representative coiled-coil structures for the indicated protein pairs are shown. Models were generated using AlphaFold2, and the heptad register (a-g positions) was assigned using Socket2. Hydrophobic residues are colored in red and charged residues in blue (all other residues in gray), highlighting the distribution of the hydrophobic core and electrostatic features across different pairings.

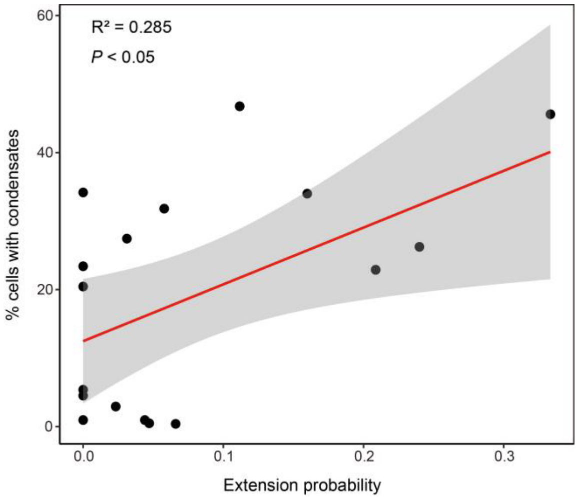

Supplemental Figure S9. Relationship between extension probability and condensate formation across CCDs. Each point represents one CCD, plotted by its extension probability and the percentage of cells with condensates. The red line shows the linear regression fit, with the shaded region indicating the 95% confidence interval. A modest but statistically significant positive association was observed between extension probability and condensate formation ($R^2 = 0.285$, $P < 0.05$).

Supplemental Table S1. Full length fusion protein amino acid sequences with both partial genes and CCDs highlighted in different colors. Red color denotes partial gene A. Blue color denotes partial gene B. Yellow highlighted the CCD regions in this study.

Fusion Proteins	Amino acid sequence
**CCDC6-PCBD1**	MADSASESDTDGAGGNSSSSAAMQSSCSSTSGGGGGGGGGGGGGKSGGIVISPFRLEELTNRLASL<br>QQENKVLKIELETYKLKCKALQEENRDLRKASVTIAGKAHRLSAEERDQLLPNLRAVGWNELEGRD<br>AIFKQFHFKDFNRAFGFMTRVALQAEKLDHHPEWFNVYNKVHITLSTHECAGLSERDINLASFIEQ<br>VAVSMT
**CXCL16-TEKT1**	MSGSQSEVAPSPQSPRSPEMGRDLRPGSRVLLLLLLLLLVYLTQPGNGNEGSVTGSCYCGKRISSD<br>SPPSVQFMNRLRKHLRAYHRCLYYTRFQLLSWSVCGGNKDPWVQELMSCLDLKEQRLEEVQFWKKE<br>LDDKLEQLVNVTDDLLIYKIRLEKALETLKEPLHITETCLAYREKRIGIDLVHDTVEHELIKEAEI<br>IQGIMALLTRTLEEASEQIRMNRSAKYNLEKDLKDKFVALTIDDICFSLNNNSPNIRYSENAVRIE<br>PNSVSLEDWLDFSSTNVEKADKQRNNSLMLKALVDRILSQTANDLRKQCDVVDTAFKNGLKDTKDA<br>RDKLADHLAKVMEEIASQEKNITALEKAILDQEGPAKVAHTRLETRTHRPNVELCRDVAQYRLMKE<br>VQEITHNVARLKETLAQAQAELKGLHRRQLALQEEIQVKENTIYIDEVLCMQMRKSIPLRDGEDHG<br>VWAGGLRPDAVC
**DVL2-TFE3**	MAGSSTGGGGVGETKVIYHLDEEETPYLVKIPVPAERITLGDFKSVLQRPAGAKYFFKSMDQDFGV<br>VKEEISDDNARLPCFNGRVVSWLVSSDNPQPEMAPPVHEPRAELAPPAPPLPPLPPERTSGIGDSR<br>PPSFHPNVSSSHENLEPETETESVVSLRRERPRRRDSSEHGAGGHRTGGPSRLERHLAGYESSSTL<br>MTSELESTSLGDSDEEDTMSSLPISLQATPATPATLSASSSAGGSRTPAMSSSSSSRVLLRQQLMR<br>AQAQEQERRERREQAAAAPFPSPAPASPAISVVGVSAGGHTLSRPPPAQVPREVLKVQTHLENPTR<br>YHLQQARRQQVKQYLSTTLGPKLASQALTPPPGPASAQPLPAPEAAHTTGPTGSAPNSPMALLTIG<br>SSSEKEIDDVIDEIISLESSYNDEMLSYLPGGTTGLQLPSTLPVSGNLLDVYSSQGVATPAITVSN<br>SCPAELPNIKREISETEAKALLKERQKKDNHNLIERRRRFNINDRIKELGTLIPKSSDPEMRWNKG<br>TILKASVDYIRKLQKEQQRSKDLESRQRSLEQANRSLQLRIQELELQAQIHGLPVPPTPGLLSLAT<br>TSASDSLKPEQLDIEEEGRPGAATFHVGGGPAQNAPHQQPPAPPSDALLDLHFPSDHLGDLGDPFH<br>LGLEDILMEEEEGVVGGLSGGALSPLRAASDPLLSSVSPAVSKASSRRSSFSMEEES
**GYS1-HOMER3**	MPLNRTLSMSSLPGLEDWEDEFDLENAVLFEVAWEVANKVGGIYTVLQTKAKVTGDEWGDNYFLVG<br>PYTEQGVRTQVELLEAPTPALKRTLDSMNSKGCKFAEKFQEVKEAARLAREKSQDGGELTSPALGL<br>ASHQVPPSPLVSANGPGEEKLFRSQSADAPGPTERERLKKMLSEGSVGEVQWEAEFFALQDSNNKL<br>AGALREANAAAAQWRQQLEAQRAEAERLRQRVAELEAQAASEVTPTGEKEGLGQGQSLEQLEALVQ<br>TKDQEIQTLKSQTGGPREALEAAEREETQQKVQDLETRNAELEHQLRAMERSLEEARAERERARAE<br>VGRAAQLLDVRLFELSELREGLARLAEAAP
**LAMA4-PSAP**	MALSSAWRSVLPLWLLWSAACSRAASGDDNAFPFDIEGSSAVGRQDPPETSEPRVALGRLPPAAEK<br>CNAGFFHTLSGECVPCDCNGNSNECLDGSGYCVHCQRNTTGEHCEKCLDGYIGDSIRGAPQFCQPC<br>PCPLPHLANFAESCYRKNGAVRCICNENYAGPNCERCAPGYYGNPLLIGSTCKKCDCSGNSDPNLI<br>FEDCDEVTGQCRNCLRNTTGFKCERCAPGYYGDARIAKNCAVCNCGGGPCDSVTGECLEEGFEPPT<br>GCDKCVWDLTDDLRLAALSIEEGKSGVLSVSSGAAAHRHVNEINATIYLLKTKLSERENQYALRKI<br>QINNAENTMKSLLSDVEELVEKENQASRKGQLVQKESMDTINHASQLVEQAHDMRDKIQEINNKML<br>YYGEEHELSPKEISEKLVLAQKMLEEIRSRQPFFTQRELVDEEADEAYELLSQAESWQRLHNETRT<br>LFPVVLEQLDDYNAKLSDLQEALDQALNHVRDAEDMNRATAARQRDHEKQQPKEICALVGFCDEVK<br>EMPMQTLVPAKVASKNVIPALELVEPIKKHEVPAKSDVYCEVCEFLVKEVTKLIDNNKTEKEILDA<br>FDKMCSKLPKSLSEECQEVVDTYGSSILSILLEEVSPELVCSMLHLCSGTRLPALTVHVTQPKDGG<br>FCEVCKKLVGYLDRNLEKNSTKQEILAALEKGCSFLPDPYQKQCDQFVAEYEPVLIEILVEVMDPS<br>FVCLKIGACPSAHKPLLGTEKCIWGPSYWCQNTETAAQCNAVEHCKRHVWN
**MED1-KRT222**	MKAQGETEESEKLSKMSSLLERLHAKFNQNRPWSETIKLVRQVMLEEDISKKMDKDEEALKAAQAE<br>LKEARRQWHHLQVEIESLHAVERGLENSLHASEQHYQMQLQDLETVIEGLEKELQEVRRGIEKQLQ<br>EHEMLLNTKMRLEQEIATYRHLLEKEEIRYYGCIQGGKKDKKPTTSRVGFVLPSAIINEISFTTKV

	PQKYENENVETVTKQAILNGSIVKESTEAHGTIQTEKVDEVIKEWEGSFFKDNPRLRKKSVSLRFD<br>LHLAATDEGCLETKQDNLPDIEVRLIMRRSCSIPSIKPPSTAN
**NSFL1C-HOOK2**	MAAERQEALREFVAVTGAEEDRARFFLESAGWDLQLQTFHVPSPCASPQDLSSGLAVAYVLNQIDP<br>SWFNEAWLQGISEDPGPNWKLKVSNLKMVLRSLVEYSQDVLAHPVSEEHLPDVSLIGEFSDPAELG<br>KLLQLVLGCAISCEKKQDHIQRIMTLEESVQHVVMEAIQELMTKDTPDSLSPETYGNFDSQSRRYY<br>FLSEEAEEGDELQQRCLDLERQLMLLSEEKQSLAQENAGLRERMGRPEGEGTPGLTAKKLLLLQSQ<br>LEQLQEENFRLESGREDERLRCAELEREVAELQHRNQALTSLAQEAQALKDEMDELRQSSERAGQL<br>EATLTSCRRRLGELRELRRQVRQLEERNAGHAERTRQLEDELRRAGSLRAQLEAQRRQVQELQGQR<br>QEEAMKAEKWLFECRNLEEKYESVTKEKERLLAERDSLREANEELRCAQLQPRGLTQADPSLDPTS<br>TPVDNLAAEILPAELRETLLRLQLENKRLCRQEAADRERQEELQRQLEDANRARHGLETQHRLNQQ<br>QLSELRAQVEDLQKALQEQGGKTEDSILLKRKLEEHLQKLHEADLELQRKREYIEELEPPTDSSTA<br>RRIEELQHNLQKKDADLRAMEERYRRYVDKARMVMQTMEPKQRPAAGAPPELHSLRTQLRERDVRI<br>RHLEMDFEKSRSQREQEEKLLISAWYNMGMALQQRAGEERAPAHAQSFLAQQRLATNSRRGPLGRL<br>ASLNLRPTDKH
**PAX8-NFE2L2**	MPHNSIRSGHGGLNQLGGAFVNGRPLPEVVRQRIVDLAHQGVRPCDISRQLRVSHGCVSKILGRYY<br>ETGSIRPGVIGGSKPKVATPKVVEKIGDYKRQNPTMFAWEIRDRLLAEGVCDNDTVPSVSSINRII<br>RTKVQQPFNLPMDSCVATKSLSPGHTLIPSSAVTPPESPQSDSLGSTYSINGLLGIAQPGSDKRKM<br>DDSDQDSCRLSIDSQSSSSGPRKHLRTDAFSQHHLEPLECPFERQHYPEAYASPSHTKGEQVSSAT<br>FQSLVPDIPGHIESPVFIATNQAQSPETSVAQVAPVDLDGMQQDIEQVWEELLSIPELQCLNIEND<br>KLVETTMVPSPEAKLTEVDNYHFYSSIPSMEKEVGNCSPHFLNAFEDSFSSILSTEDPNQLTVNSL<br>NSDATVNTDFGDEFYSAFIAEPSISNSMPSPATLSHSLSELLNGPIDVSDLSLCKAFNQNHPESTA<br>EFNDSDSGISLNTSPSVASPEHSVESSSYGDTLLGLSDSEVEELDSAPGSVKQNGPKTPVHSSGDM<br>VQPLSPSQGQSTHVHDAQCENTPEKELPVSPGHRKTPFTKDKHSSRLEAHLTRDELRAKALHIPFP<br>VEKIINLPVVDFNEMMSKEQFNEAQLALIRDIRRRGKNKVAAQNCRKRKLENIVELEQDLDHLKDE<br>KEKLLKEKGENDKSLHLLKKQLSTLYLEVFSMLRDEDGKPYSPSEYSLQQTRDGNVFLVPKSKKPD<br>VKKN
**PCBP4-POC1A**	MSGSDGGLEEEPELSITLTLRMLMHGKEVGSIIGKPEVDFPVPPGRGRSVESVQSQPQEPVSVPQT<br>LTSTLEHIVGQLDVLTQTVSILEQRLTLTEDKLKQCLENQQLIMQRATP
**PDZRN3-FOXP1**	MGFELDRFDGDVDPDLKCALCHKVLEDPLTTPCGHVFCAGCVLPWVVQEGSCPARCRGRLSAKELN<br>HVLPLKRLILKLDIKCAYATRGCGRVVKLQQLPEHLERCDFAPARCRHAGCGQVLLRRDVEAHMRD<br>ACDARPVGRCQEGCGLPLTHGEQRAGGHCCARALRAHNGALQARLGALHKALKKEALRAGKREKSL<br>VAQLAAAQLELQMTALRYQKKFTEYSARLDSLSRCVAAPPGGKGEETKSLTLVLHRDSGSLGFNII<br>GGRPSVDNHDGSSSEGIFVSKIVDSGPAAKEGGLQIHDRIIEVPVSVAMMTPQVITPQQMQQILQQ<br>QVLSPQQLQVLLQQQQALMLQQQQLQEFYKKQQEQLQLQLLQQQHAGKQPKEQQQVATQQLAFQQQ<br>LLQMQQLQQQHLLSLQRQGLLTIQPGQPALPLQPLAQGMIPTELQQLWKEVTSAHTAEETTGNNHS<br>SLDLTTTCVSSSAPSKTSLIMNPHASTNGQLSVHTPKRESLSHEEHPHSHPLYGHGVCKWPGCEAV<br>CEDFQSFLKHLNSEHALDDRSTAQCRVQMQVVQQLELQLAKDKERLQAMMTHLHVKSTEPKAAPQP<br>LNLVSSVTLSKSASEASPQSLPHTPTTPTAPLTPVTQGPSVITTTSMHTVGPIRRRYSDKYNVPIS<br>SADIAQNQEFYKNAEVRPPFTYASLIRQAILESPEKQLTLNEIYNWFTRMFAYFRRNAATWKNAVR<br>HNLSLHKCFVRVENVKGAVWTVDEVEFQKRRPQKISGNPSLIKNMQSSHAYCTPLNAALQASMAEN<br>SIPLYTTASMGNPTLGNLASAIREELNGAMEHTNSNESDSSPGRSPMQAVHPVHVKEEPLDPEEAE<br>GPLSLVTTANHSPDFDHDRDYEDEPVNEDME
**SPAG9-SKAP1**	MELEDGVVYQEEPGGSGAVMSERVSGLAGSIYREFERLIGRYDEEVVKELMPLVVAVLENLDSVFA<br>QDQEHQVELELLRDDNEQLITQYEREKALRKHAEEKFIEFEDSQEQEKKDLQTRVESLESQTRQLE<br>LKAKNYADQMEDIVKGAQELDNVIKQGYLEKKSKDHSFFGSEWQKRWCVVSRGLFYYYANEKSKQP<br>KGTFLIKGYGVRMAPHLRRDSKKESCFELTSQDRRSYEFTATSPAEARDWVDQISFLLKDLSSLTI<br>PYEEDEEEEEKEETYDDIDGFDSPSCGSQCRPTILPGSVGIKEPTEEKEEEDIYEVLPDEEHDLEE<br>DESGTRRKGDYASYYQGLWDCHGDQPDELSFQRGDLIRILSKEYNMYGWWVGELNSLVGIVPKEYL<br>TTAFEVEER
**STRN3-METTL3**	MDELAGGGGGGPGMAAPPRQQQGPGGNLGLSPGGNGAAGGGGPPASEGAGPAAGPELSRPQQYTIP<br>GILHYIQHEWARFEMERAHWEVERAELQARIAFLQGERKGQENLKKDLVRRIKMLEYALKQERAKY<br>HKLKYGTELNQGDLKMPTFESEETKDTEAPTAPQNSQLTWKQGRQLLRQYLQEVGYTDTILDVRSQ<br>RVRSLLGLSNSEPNGSVETKNLEQILNGGESPKQKGQEIKRSSGDVLETFNFLENADDSDEDEEND<br>MIEGIPEGKDKHRMNKHKIGNEGLAADLTDDPDTEEALKEFDFLVTAEDGEGAGEARSSGDGTEWD<br>LRNPEAALSPTFRSDSPVPTAPTSGGPKPSTASAVPELATDPELEKKLLHHLSDLALTLPTDAVSI

	CLAISTPDAPATQDGVESLLQKFAAQELIEVKRGLLQDDAHPTLVTYADHSKLSAMMGAVAEKKGP<br>GEVAGTVTGQKRRAEQDSTTVAAFASSLVSGLNSSASEPAKEPAKKSRKHAASDVDLEIESLLNQQ<br>STKEQQSKKVSQEILELLNTTTAKEQSIVEKFRSRGRAQVQEFCDYGTKEECMKASDADRPCRKLH<br>FRRIINKHTDESLGDCSFLNTCFHMDTCKYVHYEIDACMDSEAPGSKDHTPSQELALTQSVGGDSS<br>ADRLFPPQWICCDIRYLDVSILGKFAVVMADPPWDIHMELPYGTLTDDEMRRLNIPVLQDDGFLFL<br>WVTGRAMELGRECLNLWGYERVDEIIWVKTNQLQRIIRTGRTGHWLNHGKEHCLVGVKGNPQGFNQ<br>GLDCDVIVAEVRSTSHKPDEIYGMIERLSPGTRKIELFGRPHNVQPNWITLGNQLDGIHLLDPDVV<br>ARFKQRYPDGIISKPKNL
**TRIM27-PTPRR**	MASGSVAECLQQETTCPVCLQYFAEPMMLDCGHNICCACLARCWGTAETNVSCPQCRETFPQRHMR<br>PNRHLANVTQLVKQLRTERPSGPGGEMGVCEKHREPLKLYCEEDQMPICVVCDRSREHRGHSVLPL<br>EEAVEGFKEQIQNQLDHLKRVKDLKKRRRAQGEQARAELLSLTQMEREKIVWEFEQLYHSLKEHEY<br>RLLARLEELDLAIYNSINGAITQFSCNISHLSSLIAQLEEKQQQPTRELLQDIGDTLSSAGIGRTG<br>CFIATSIGCQQLKEEGVVDALSIVCQLRMDRGGMVQTSEQYEFVHHALCLYESRLSAETVQ
**VPS37B-MPHOSPH9**	MAGAGSEARFAGLSLVQLNELLEDEGQLTEMVQKMEELLGEYESLGKEHRRVKDALNTTENKLLDA<br>YTQISDLKRMISKLEAQVKQVEHENMLSLRHNSRIHVRPSRANTLATSDVSRRKWLIPGAEYSIFT<br>GQPLDTQDSNVDNQLEETCSLGHRSPLEKDSSPGSSSTSLLIKKQRETSDTPIMRALKELDEGKIF<br>KNWGTQTEKEDTSNINPRQTETSVNASRSPEKCAQQRQKRLNSASQRSSSLPPSNRKSSTPTKREI<br>MLTPVTVAYSPKRSPKENLSPGFSHLLSKNESSPIRFDILLDDLDTVPVSTLQRTNPRKQLQFLPL<br>DDSEEKTYSEKATDNHVNHSSCPEPVPNGVKKVSVRTAWEKNKSVSYEQCKPVSVTPQGNDFEYTA<br>KIRTLAETERFFDELTKEKDQIEAALSRMPSPGGRITLQTRLNQEALEDRLERINRELGSVRMTLK<br>KFHVLRTSANL

Supplemental Table S2. List of amino acid sequence of predicted CCDs

FP-CCDs	Amino acid sequences
**CCDC6-PCBD1**	LASLQQENKVLKIELETYKLKCKALQEENRDLRKA
**CXCL16-TEKT1$_3$**	QYRLMKEVQEITHNVARLKETLAQAQAELKGLHRRQLALQEEIQVKENT
**DVL2-TFE3**	SVDYIRKLQKEQQRSKDLESRQRSLEQANRSLQLR
**GYS1-HOMER3$_1$**	NNKLAGALREANAAAAQWRQQLEAQRAEAERLRQR
**GYS1-HOMER3$_2$**	REETQQKVQDLETRNAELEHQLRAMERSLEEARAERERARAEVGRAAQL LDVRLFELSELREG
**LAMA4-PSAP$_1$**	VNEINATIYLLKTKLSERENQYALRKIQINNAENTMKSLLSDVEELVEK
**LAMA4-PSAP$_2$**	AYELLSQAESWQRLHNETRTLFPVVLEQLDDYNAKLSDLQEALDQALNH
**MED1-KRT222**	VMLEEDISKKMDKDEEALKAAQAELKEARRQWHHLQVEIESLHAVERGL ENSLHASEQHYQMQLQDLETVIEGLEKELQEVRRGIEKQLQEHEMLLNT KMRLEQEIATYRHL
**NSFL1C-HOOK2$_1$**	LEQLQEENFRLESGREDERLRCAELEREVAELQHRNQALTSLAQEAQAL KDEMDEL
**NSFL1C-HOOK2$_2$**	AGSLRAQLEAQRRQVQELQGQRQEEAMKAEKWLFECRNLEEKYESVTKE KERLLAERDSLREA
**NSFL1C-HOOK2$_3$**	LLRLQLENKRLCRQEAADRERQEELQRQLEDANRARHGLETQHRLNQQQ LSELRAQVEDLQKA
**PAX8-NFE2L2**	IVELEQDLDHLKDEKEKLLKEKGENDKSLHLLKKQLSTLYLE
**PCBP4-POC1A**	LEHIVGQLDVLTQTVSILEQRLTLTEDKLKQCLEN

**PDZRN3-FOXP1**	QARLGALHKALKKEALRAGKREKSLVAQLAAAQLELQMTALRYQKKFTE
**SPAG9-SKAP1**	VVAVLENLDSVFAQDQEHQVELELLRDDNEQLITQYEREKALRKHAEEK FIEFEDSQEQEKKDLQTRVESLESQTRQLELKAKN
**STRN3-METTL3**	RAELQARIAFLQGERKGQENLKKDLVRRIKMLEYA
**TRIM27-PTPRR**	DHLKRVKDLKKRRRAQGEQARAELLSLTQMEREKIVWEFEQLYHSLKEH EYRLLARLEELDLAIYNSINGAITQFSCNISHLSS
**VPS37B-MPHOSPH9**	LTEMVQKMEELLGEYESLGKEHRRVKDALNTTENKLLDAYTQISDLKRM ISKLEAQ

Supplemental Table S3. Oligomeric state predictions of various coiled-coil domains (CCDs) based on various computational prediction tools. “x” indicates no predicted oligomer formation.

Name of CCD	CoCoPRED	CoCoNet	Marcoil	Multicoil2	Ncoils	Paircoil2
**CCDC6-PCBD1**	dimer	x	dimer	dimer	dimer	dimer
**CXCL16-TEKT1$_3$**	dimer	dimer	dimer	dimer	dimer	dimer
**DVL2-TFE3**	dimer	x	trimer	trimer	trimer	trimer
**GYS1-HOMER3$_1$**	dimer	x	dimer	x	dimer	dimer
**GYS1-HOMER3$_2$**	dimer	dimer	dimer	dimer	dimer	dimer
**LAMA4-PSAP$_1$**	dimer	dimer	dimer	x	trimer	dimer
**LAMA4-PSAP$_2$**	dimer	tetramer	trimer	x	trimer	trimer
**MED1-KRT222**	dimer	dimer	dimer	dimer	dimer	dimer
**NSFL1C-HOOK2$_1$**	dimer	dimer	dimer	dimer	dimer	dimer
**NSFL1C-HOOK2$_2$**	dimer	dimer	dimer	dimer	dimer	dimer
**NSFL1C-HOOK2$_3$**	dimer	dimer	dimer	dimer	dimer	dimer
**PAX8-NFE2L2**	dimer	dimer	dimer	dimer	dimer	dimer
**PCBP4-POC1A**	trimer	x	x	x	dimer	trimer
**PDZRN3-FOXP1**	trimer	dimer	x	x	x	x
**SPAG9-SKAP1**	dimer	dimer	dimer	dimer	dimer	dimer
**STRN3-METTL3**	dimer	x	dimer	x	dimer	dimer
**TRIM27-PTPRR**	dimer	dimer	x	x	dimer	x

**VPS37B-MPHOSPH9**	dimer	dimer	dimer	dimer	dimer	dimer

Supplemental Table S4. Mutations introduced into the coiled-coil domains (CCDs) of fusion proteins to assess their role in driving liquid-liquid phase separation (LLPS)

CCDs	Mutation
**GYS1-HOMER3$_1$**	A7E; N12E; A14K
**PAX8-NFE2L2**	L19K; S28K; Y40E
**NSFL1C_HOOK2$_1$**	G14K; L42K; Q47K; L56E
**NSFL1C-HOOK2$_2$**	Q19E; L54K; A63E
**LAMA4-PSAP$_2$**	L5E; Q12K; Q28K; A42E
**DVL2-TFE3**	Y4E; Q9E; Q33K
**SPAG9-SKAP1**	L5E; F12K; Q19E; Q65K
**CCDC6-PCBD1**	Q5E; E14S; R33E
**GYS1-HOMER3$_2$**	R14E; E26K
**STRN3-METTL3**	Q5K; E33K; A35K
**PDZRN3-FOXP1**	G5K; E14S; R21E; V26E; E49K
**VPS37B-MPHOSPH9**	K7E; E21K; E33S; K47E

Supplemental Table S5. Predicted nuclear localization signals (NLS) in full-length fusion proteins and their parental proteins. NLS prediction was performed using cNLS Mapper (https://nls-mapper.iab.keio.ac.jp/cgi-bin/NLS_Mapper_form.cgi).

Parental Protein	NLS	Fusion Protein	NLS
CCDC6	No	CCDC6-PCBD1	No
PCBD1	No		
CXCL16	Yes	CXCL16-TEKT1	No
TEKT1	No		
DVL2	No	DVL2-TFE3	No
TFE3	No		
GYS1	No	GYS1-HOMER3	No
HOMER3	No		
LAMA4	No	LAMA4-PSAP	No
PSAP	No		
MED1	Yes	MED1-KRT222	No
KRT222	No		
NSFL1C	No	NSFL1C-HOOK2	No
HOOK2	Yes		
PAX8	No	PAX8-NFE2L2	Yes
NFE2L2	Yes		
PCBP4	No	PCBP4-POC1A	No
POC1A	No		
PDZRN3	No	PDZRN3-FOXP1	No

FOXP1	No		
SPAG9	No	SPAG9-SKAP1	No
SKAP1	No		
STRN3	No	STRN3-METTL3	Yes
METTL3	Yes		
TRIM27	Yes	TRIM27-PTPRR	Yes
PTPRR	No		
VPS37B	No	VPS37B-MPHOSPH9	No
MPHOSPH9	No		
